# Supplementary material for: Defining essential charged residues in fibril formation of a lysosomal derived N-terminal α-synuclein truncation
Source: Nat Commun. 2025 Apr 23;16:3825. doi: 10.1038/s41467-025-58899-9 (PMC12019160; doi:10.1038/s41467-025-58899-9)
Supplement: Supplementary file 1 — Supplementary Information [file 41467_2025_58899_MOESM1_ESM.pdf]

Supporting Information for

Defining essential charged residues in fibril formation of a lysosomal derived N-terminal α-synuclein truncation

RP McGlinchey, S Ramos, EK Dimitriadis, CB Wilson, JC Lee

Supplementary Figure 1. Cathepsin B, L, D and AEP digestion of soluble α-syn

Supplementary Figure 2. Cathepsin B, L, D and AEP digestion of fibrillar α-syn

Supplementary Figure 3. A salt-dependent aggregation of 66–140

Supplementary Figure 4. Additional self-seeding data of 66–140

Supplementary Figure 5. Full TEM and AFM images of 66–140 fibrils

Supplementary Figure 6. 66–140 and Ac1–140 Raman difference spectra

Supplementary Figure 7. 2D carbon-carbon cross-polarization spectra

Supplementary Figure 8. 2D NCACX spectra of 66–140 fibrils

Supplementary Figure 9. 2D NCOCX spectra of 66–140 fibrils

Supplementary Figure 10. 3D NCACX spectra of 66–140 fibrils

Supplementary Figure 11. 3D NCOCX spectra of 66–140 fibrils

Supplementary Figure 12. 3D CANCOCX spectra of 66–140 fibrils

Supplementary Figure 13. REDOR experiment on 66–140 fibrils

Supplementary Figure 14. Complete aggregation kinetics of 66–140 and variants

Supplementary Figure 15. Full TEM and AFM images of single-Ala mutant fibrils

Supplementary Figure 16. Full Raman spectra of single-Ala mutants

Supplementary Figure 17. Additional cross-seeding data of soluble 66–140

Supplementary Figure 18. ThT kinetics of E104A/E105A seeded with 66–140 fibrils

Supplementary Figure 19. ThT kinetics of K96A/K97A and cross-seeding by 66–140 fibrils

Supplementary Figure 20. Additional ThT kinetics data of soluble single-Ala mutants seeded with 66–140 fibrils

Supplementary Figure 21. Full TEM images of single-Ala mutants seeded with 66–140 fibrils

Supplementary Figure 22. Full Raman spectra of single-Ala mutants seeded with 66–140 fibrils

Supplementary Figure 23. Full TEM image of E83K/K97E seeded with 66–140 fibrils

Supplementary Figure 24. Full Raman spectra of E83K/K97E seeded with 66–140 fibrils

Supplementary Figure 25. 66–140 model structure by molecular dynamics simulations
Supplementary Figure 26. ThT kinetics of Ac1-140 and single Ala-mutants
Supplementary Figure 27. Full TEM images of Ac1–140 and single Ala-mutants
Supplementary Figure 28. Full Raman spectra of Ac1–140 and single Ala-mutants
Supplementary Table 1. MS analysis of lysosomal digestion of soluble and fibrillar 66–140
Supplementary Table 2. MS analysis of cathepsin digestion of soluble α-syn
Supplementary Table 3. MS analysis of cathepsin digestion of fibrillar α-syn
Supplementary Table 4. List of NMR experimental parameters
Supplementary Table 5. Chemical shifts and predicted backbone torsion angles for 66–140 fibrils
Supplementary Table 6. MS analysis of PK digestion of 66–140 fibrils
Supplementary Table 7. Values of t_{lag} and $t_{½}$ for 66–140 constructs
Supplementary Table 8. MS analysis of PK digestion of single-Ala mutant fibrils

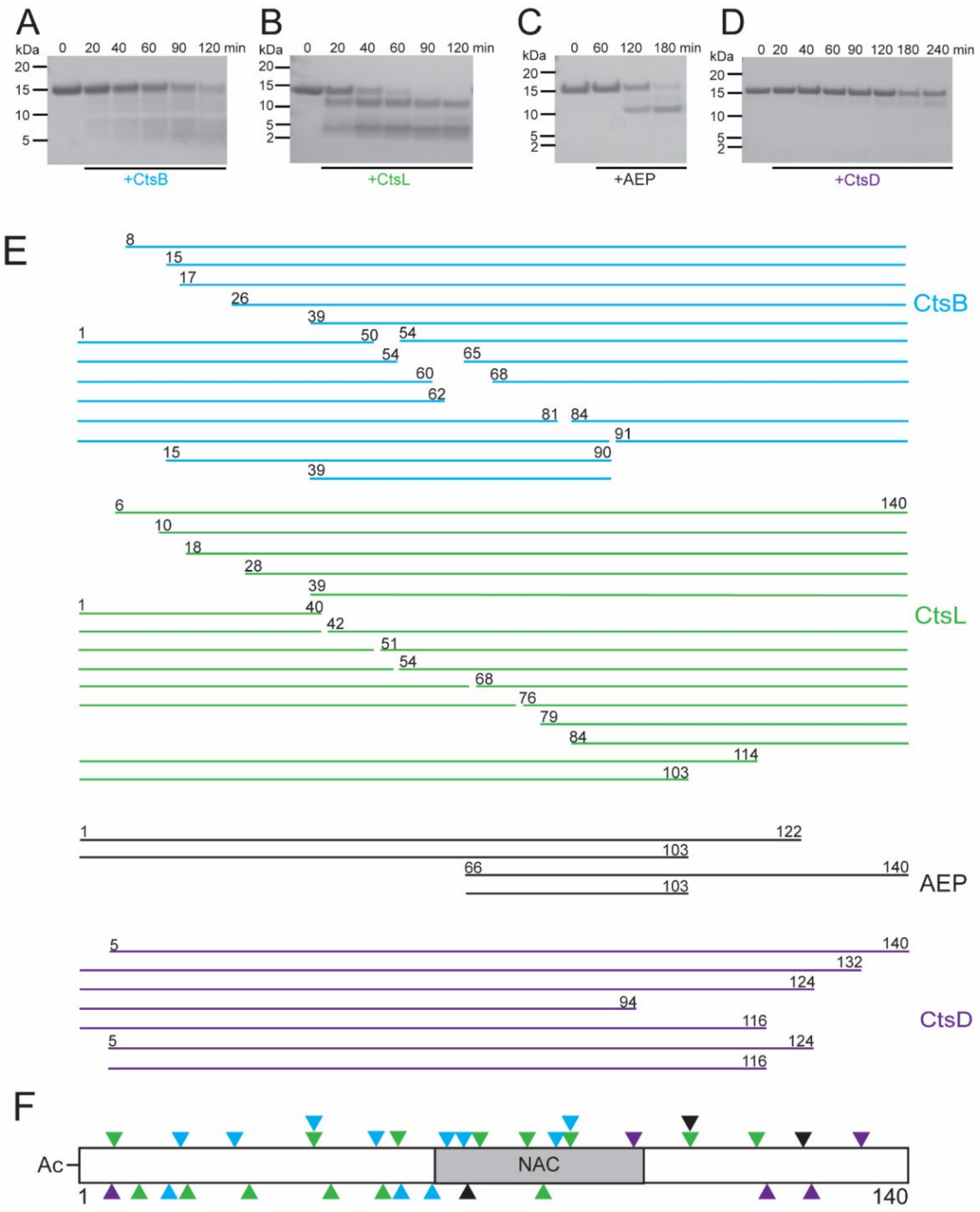

Supplementary Figure 1. CtsB, CtsL, AEP and CtsD degradation of soluble α-syn. SDS-PAGE analysis of CtsB (**A**), CtsL (**B**), AEP (**C**) and CtsD (**D**) degradation of soluble α-syn over time at pH 5. (**E**) Peptide fragments identified by LC-MS analysis from CtsB (cyan), CtsL (green), AEP (black) and CtsD (purple) activity of soluble α-syn. (**F**) Schematic representation of the primary amino acid sequence of α-syn (residues 1 to 140) with LC-MS-mapped cleavage sites for CtsB (cyan), CtsL (green), AEP (black) and CtsD (purple) generated from soluble α-syn.

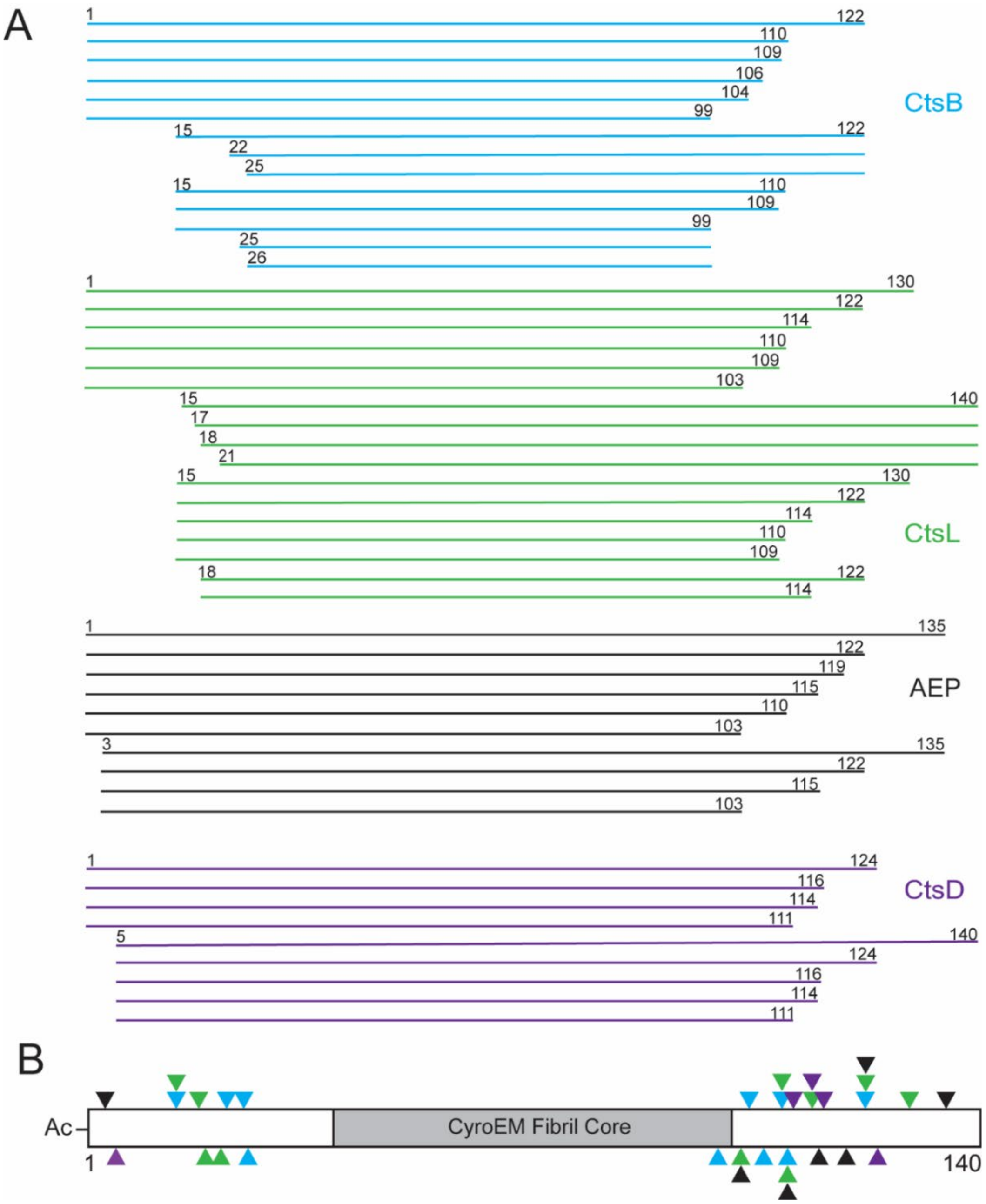

Supplementary Figure 2. CtsB, CtsL, AEP and CtsD degradation of fibrillar α-syn. (**A**) Peptide fragments identified by LC-MS analysis from CtsB (cyan), CtsL (green), AEP (black) and CtsD (purple) activity on fibrillar α-syn. (**B**) Schematic representation of the primary amino acid sequence of α-syn (residues 1 to 140) with LC-MS-mapped cleavage sites for CtsB (cyan), CtsL (green), AEP (black) and CtsD (purple) generated from fibrillar α-syn.

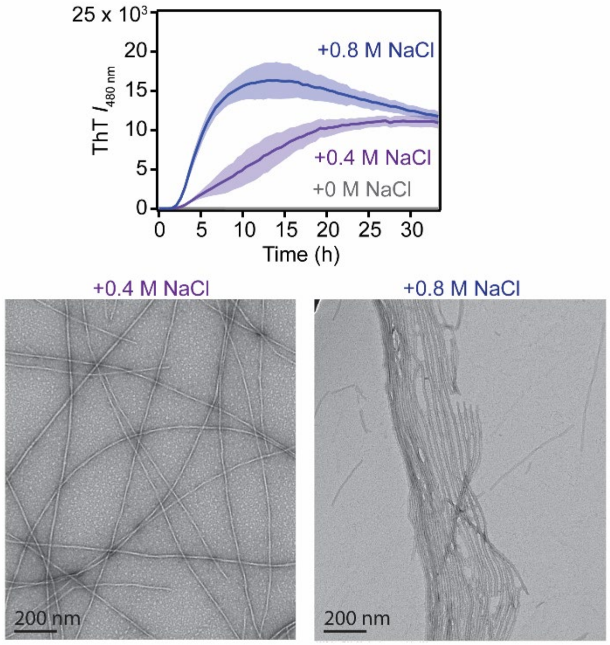

Supplementary Figure 3. Aggregation kinetics monitored by ThT (10 µM) fluorescence of soluble 66–140 (40 µM) aggregated in pH 7.4 buffer (20 mM NaPi) containing either 0 (*gray*), 0.4 (*purple*), 0.8 (*blue*) M NaCl at 37 °C with continuous linear shaking supplemented with a 2-mm glass bead. Solid lines and shaded regions represent mean and SD, respectively ($n \geq 6$). Representative TEM images ($n \geq 3$) of 66–140 fibrils taken post aggregation in 0.4 and 0.8 M NaCl. Scale bar are as shown. Here, the protein was buffer exchanged into milli-Q water, filtered through YM100 membranes, and the appropriate 10× buffer for each condition was added. Source data are provided as a Source Data file.

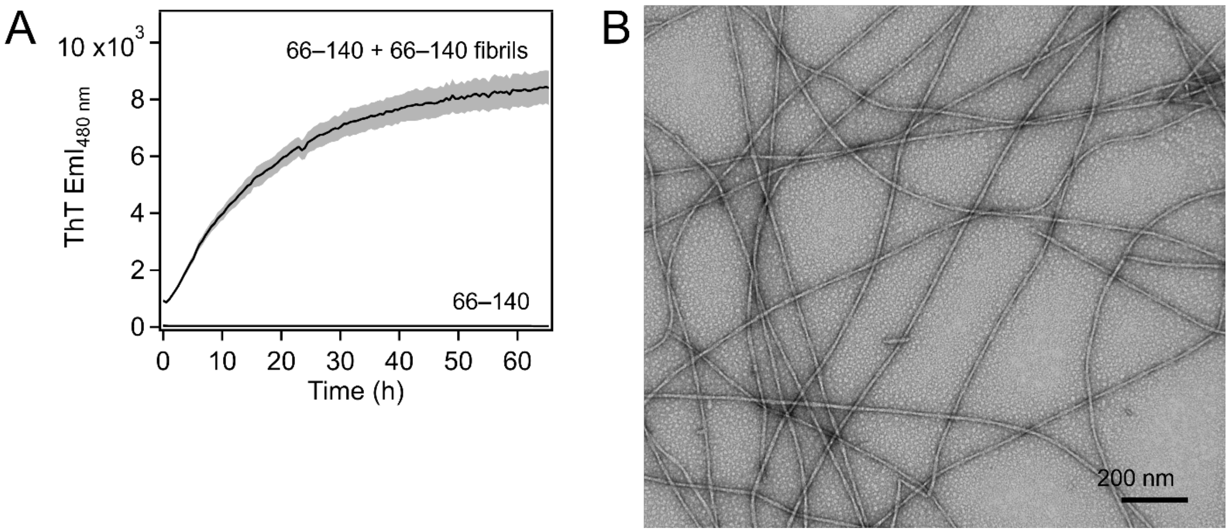

Supplementary Figure 4. (**A**) Aggregation kinetics monitored by ThT (10 μM) fluorescence of soluble 66–140 (30 μM) seeded with 66–140 fibrils (5 mol%) in pH 7.4 buffer (20 mM NaPi, 140 mM NaCl) at 37 °C with continuous linear shaking. Solid lines and shaded regions represent mean and SD, respectively (n ≥ 6). Unseeded 66–140 (30 μM) is shown as a control. (**B**) Representative TEM images (*n* = 10) of 66–140 (30 μM) seeded with 1.5 μM 66–140 fibrils. Source data are provided as a Source Data file.

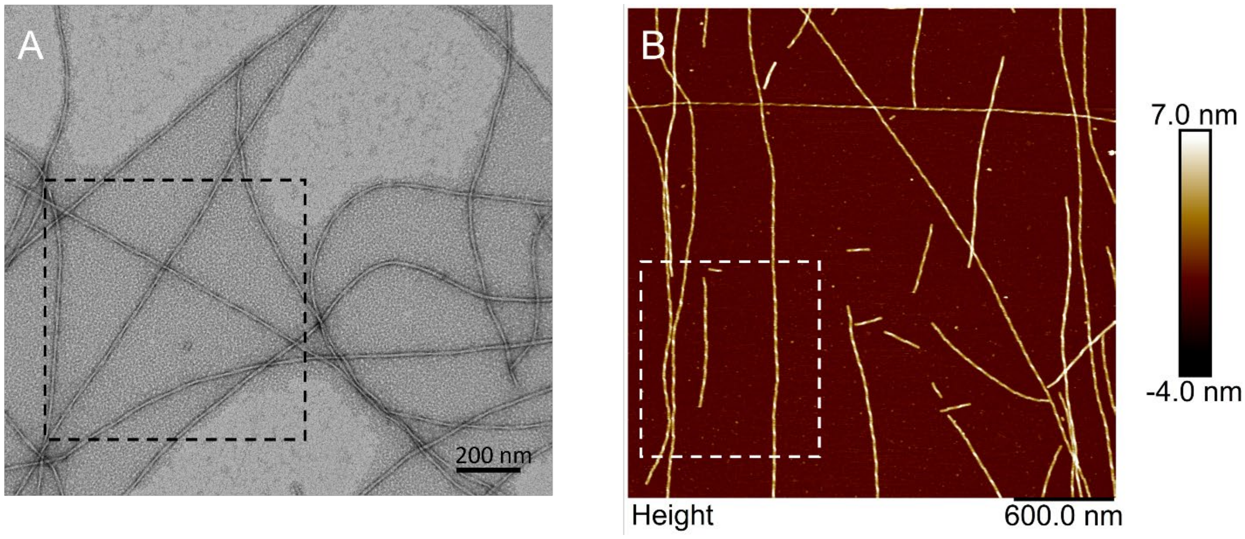

Supplementary Figure 5. Representative TEM (*n* = 7) (**A**) and AFM (*n* = 2) (**B**) images of 66–140 fibrils. Dashed areas represent images shown in Fig. 1C. Scale bars are as shown. Four fibril populations with helical pitches at 76 ± 3 nm (*n* = 8 fibrils), 85 ± 3 nm (*n* = 17 fibrils) and 103 ± 6 nm (*n* = 6 fibrils) are reported from analyzing a total of 31 fibrils by AFM. The majority of fibrils analyzed (*n* > 50 fibrils) from TEM has a ~90 nm helical pitch, which likely represents the 85 ± 3 nm observed from AFM as the main polymorph.

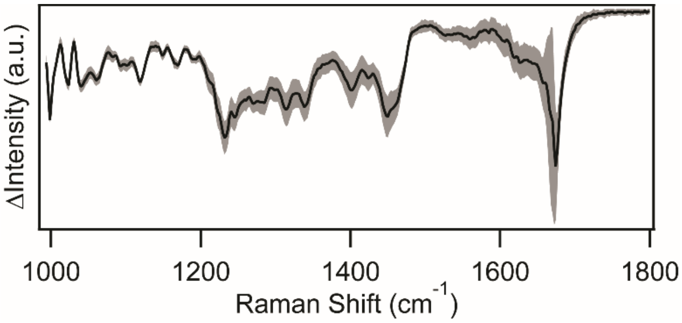

Supplementary Figure 6. 66–140 and Ac1–140 Raman Difference Spectra. The difference spectrum was generated by subtracting the spectrum of Ac1–140 from that of 66–140 (**Fig. 2**). The shaded area represents the propagated error. Source data are provided as a Source Data file.

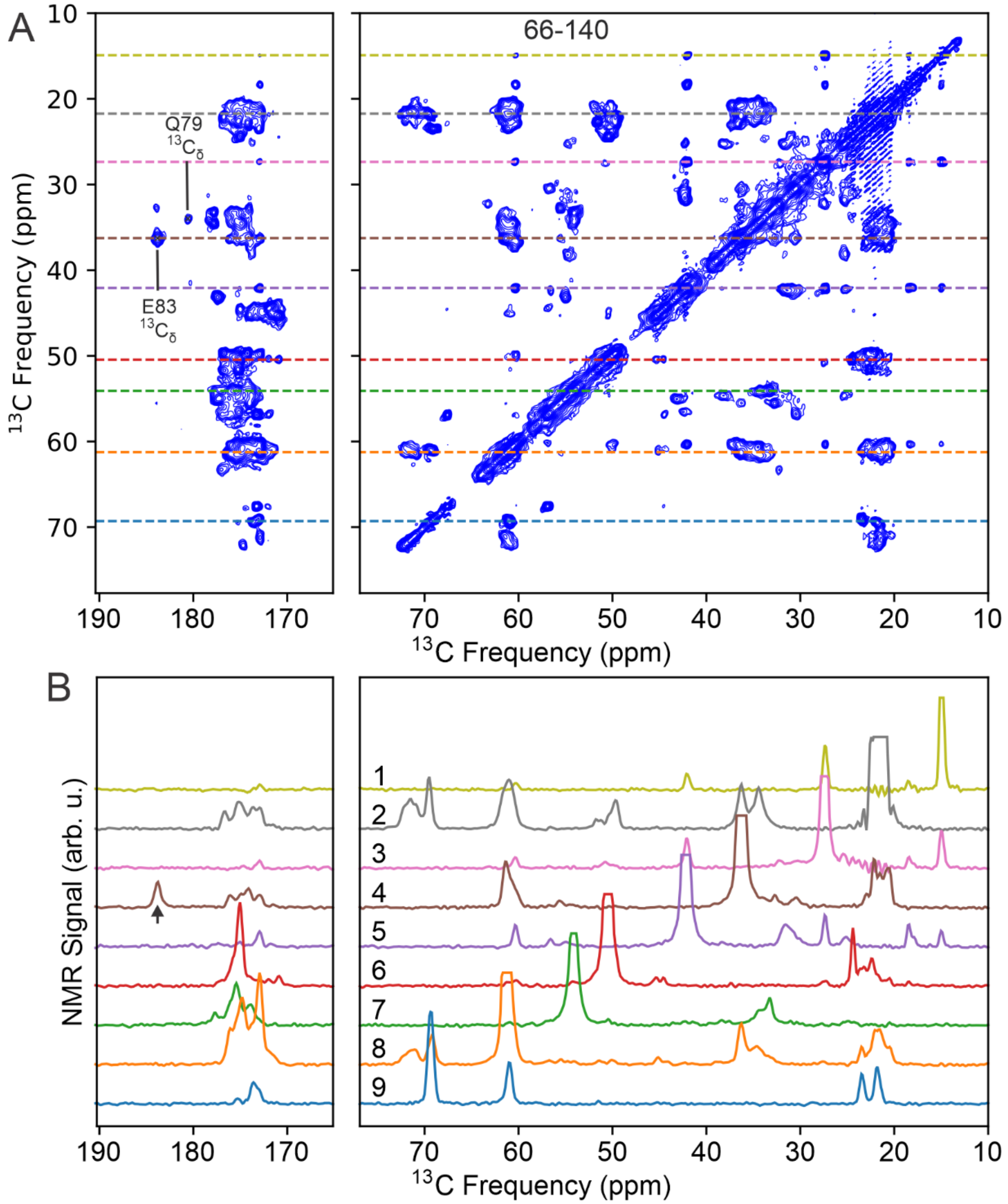

Supplementary Figure 7. 2D ^{13}C-^{13}C spectrum acquired on ^{13}C, ^{15}N-labeled 66–140 fibrils, with 17 kHz MAS. Contours are plotted with successive factors of 1.30. (**B**) 1D slices along the ^{13}C dimension at the indicated positions in the 2D ^{13}C-^{13}C spectrum. Arrow indicates E83 $^{13}C_{\delta}$ resonance. Diagonal peaks are truncated for clarity. ^{13}C FWHM linewidths were ~1 ppm, or ~180 Hz at 17.6 T. Source data are provided as a Source Data file.

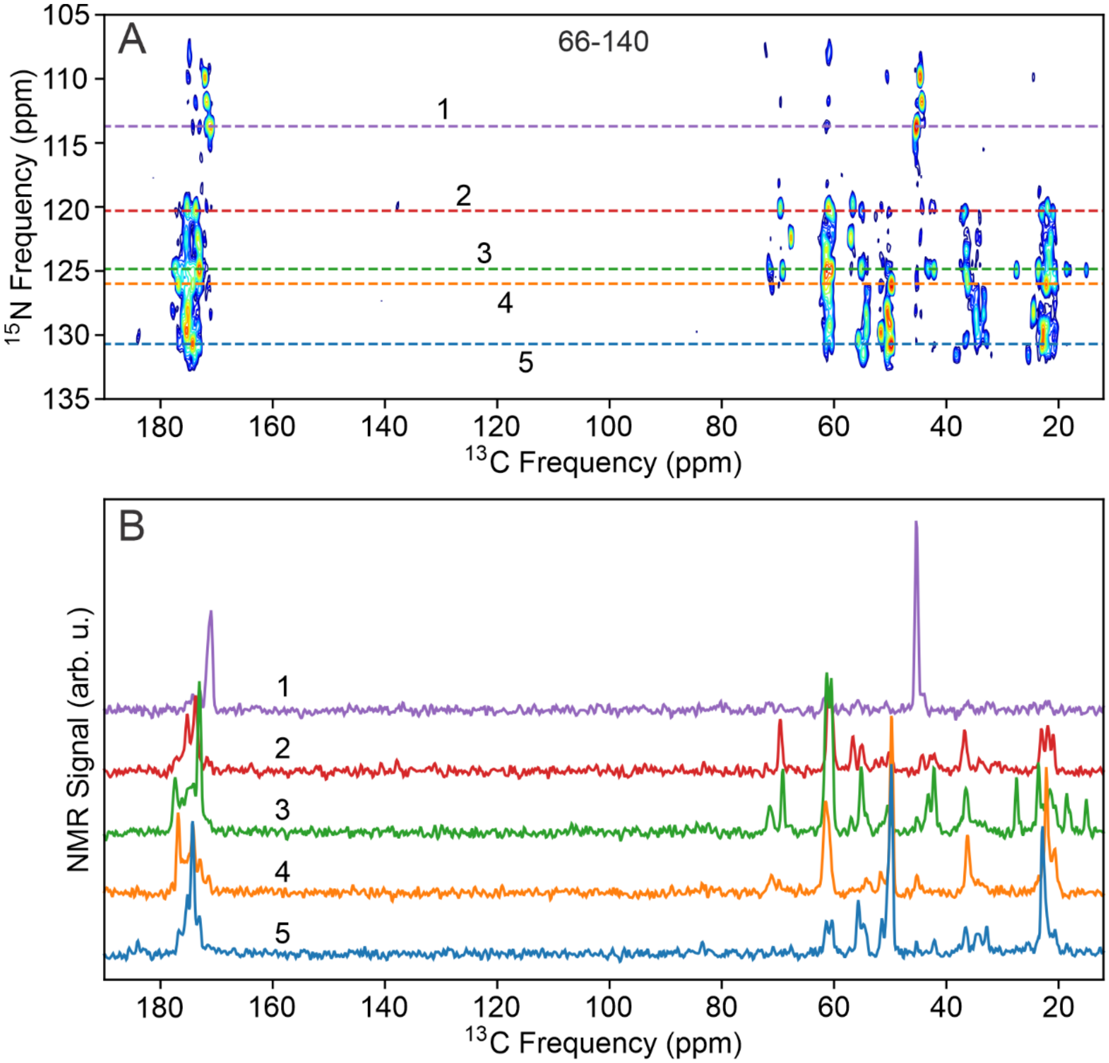

Supplementary Figure 8. (**A**) 2D NCaCX spectrum acquired on ^{13}C,^{15}N-labeled 66–140 fibrils, with 17 kHz MAS. Contours are plotted with successive factors of 1.30. (**B**) 1D slices along the ^{13}C dimension at the indicated positions in the 2D NCaCX spectrum. ^{13}C FWHM linewidths were ~1 ppm, or ~180 Hz at 17.6 T. ^{15}N FWHM linewidths were ~1.2 ppm, or ~90 Hz at 17.6 T. Source data are provided as a Source Data file.

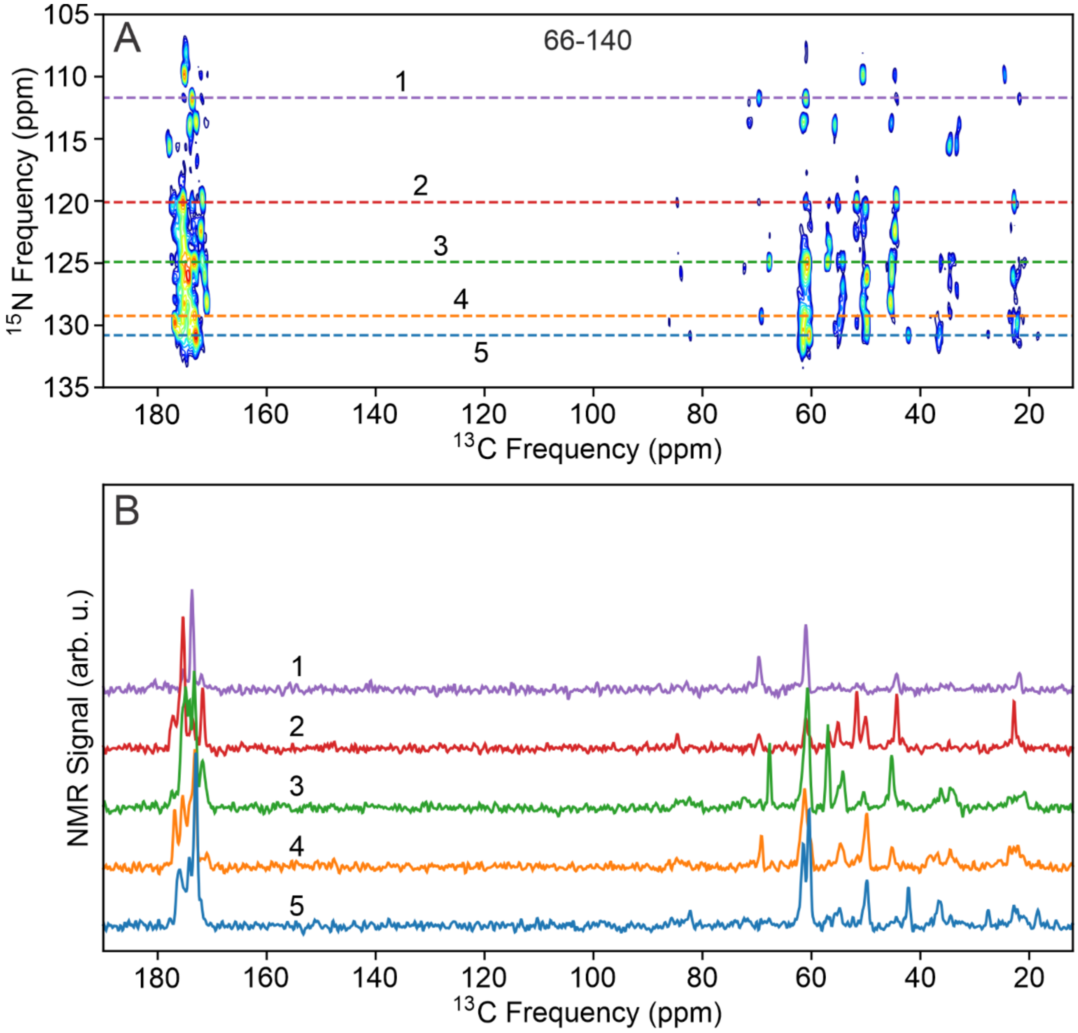

Supplementary Figure 9. (**A**) 2D NCOCX spectrum acquired on ^{13}C,^{15}N-labeled 66–140 fibrils, with 17 kHz MAS. Contours are plotted with successive factors of 1.30. (**B**) 1D slices along the ^{13}C dimension at the indicated positions in the 2D NCOCX spectrum. ^{13}C FWHM linewidths were ~1 ppm, or ~180 Hz at 17.6 T. ^{15}N FWHM linewidths were ~1.2 ppm, or ~90 Hz at 17.6 T. Source data are provided as a Source Data file.

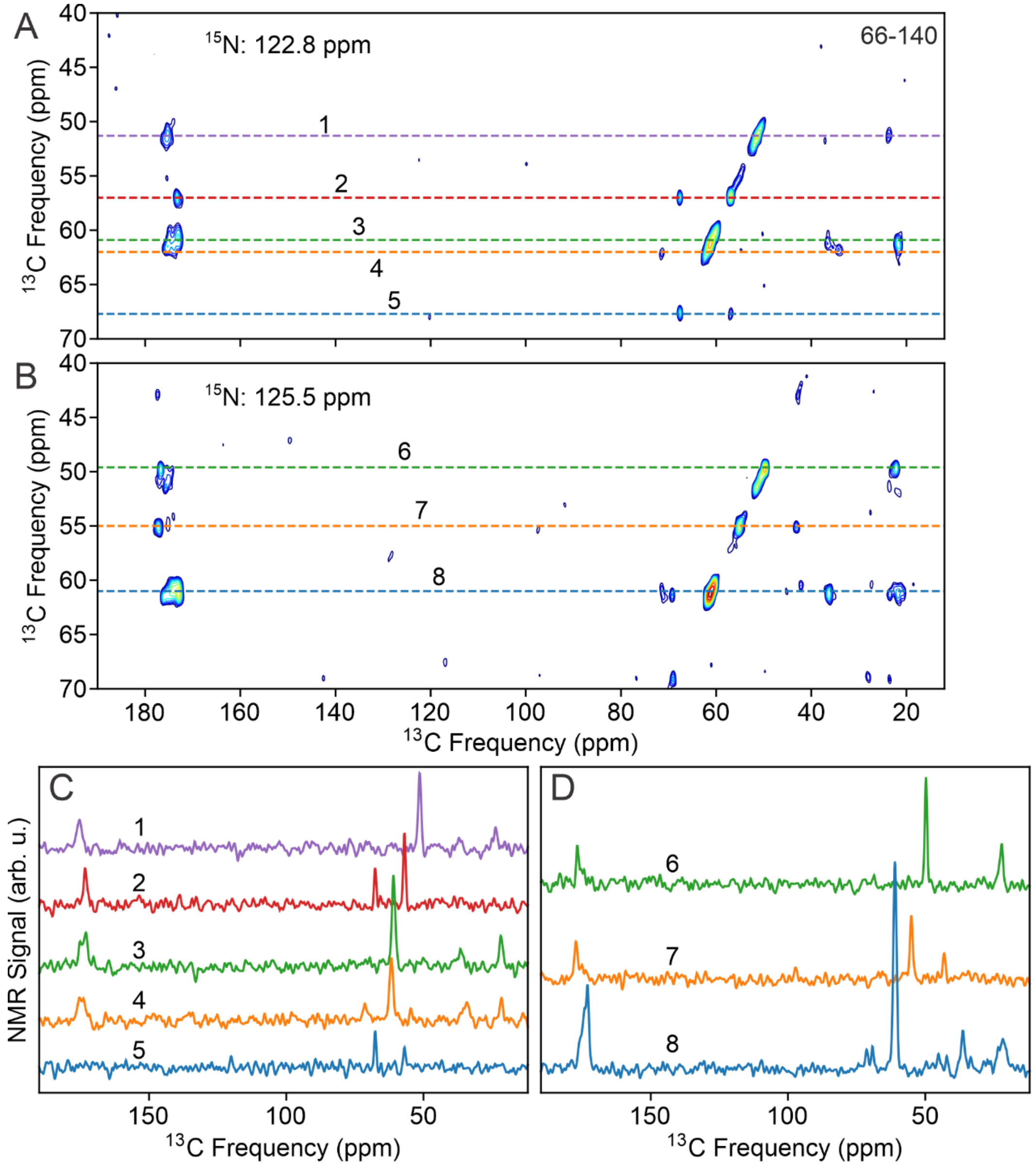

Supplementary Figure 10. 3D NCaCX spectrum acquired on ^{13}C,^{15}N-labeled 66–140 fibrils, with 17 kHz MAS. (**A**), (**B**) show representative 2D planes of constant ^{15}N frequency. Contours are plotted with successive factors of 1.30. 150 Hz Gaussian line broadening was applied in the direct and indirect ^{13}C dimensions, and 120 Hz Gaussian line broadening was applied in the indirect ^{15}N dimension. (**C**), (**D**) show 1D slices along the direct ^{13}C dimension through the 2D planes (**A**), (**B**), respectively, at the indicated positions. Source data are provided as a Source Data file.

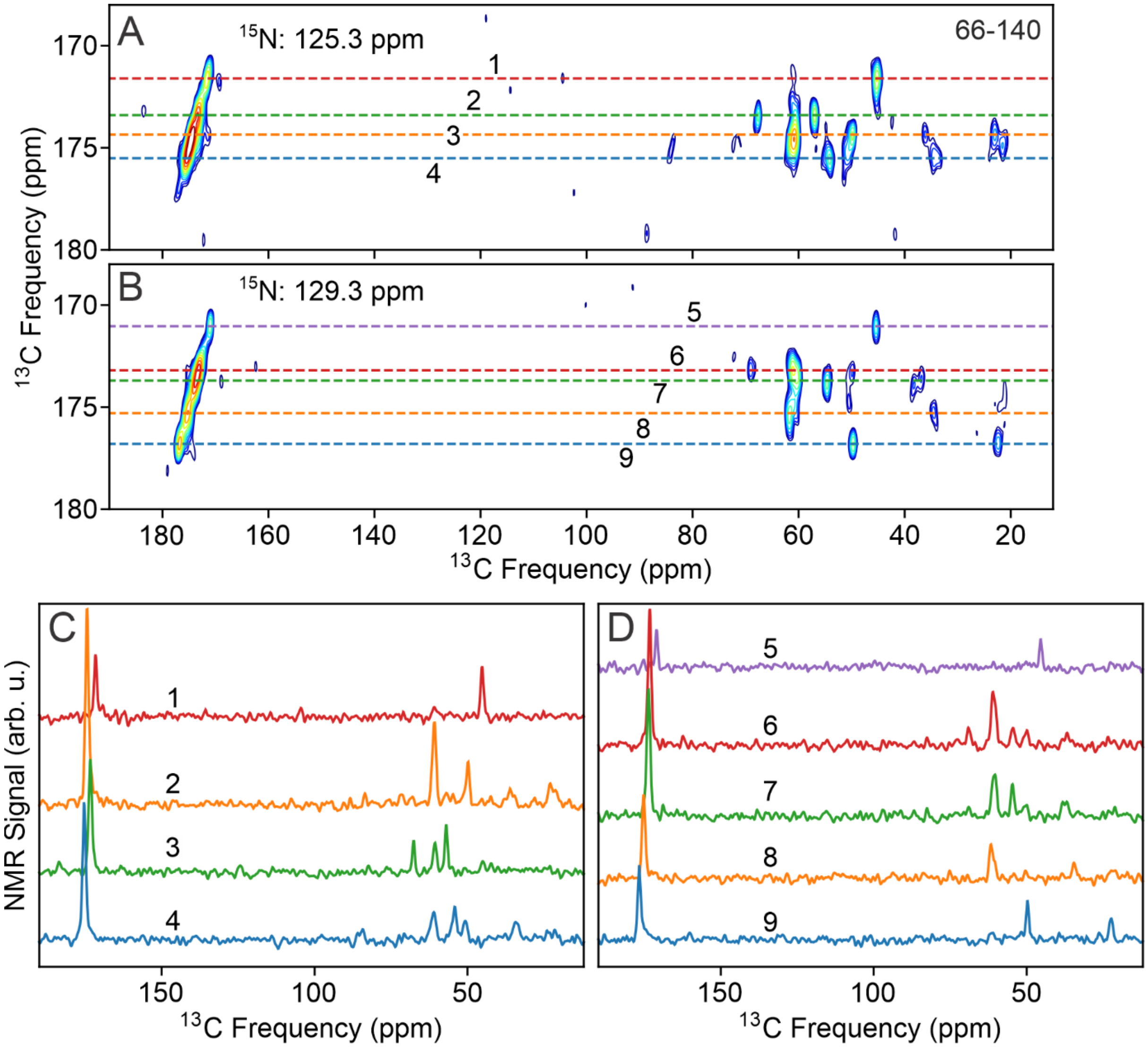

Supplementary Figure 11. 3D NCOCX spectrum acquired on ^{13}C,^{15}N-labeled 66–140 fibrils, with 17 kHz MAS. (**A**), (**B**) show representative 2D planes of constant ^{15}N frequency. Contours are plotted with successive factors of 1.30. 150 Hz Gaussian line broadening was applied in the direct and indirect ^{13}C dimensions, and 120 Hz Gaussian line broadening was applied in the indirect ^{15}N dimension. (**C**), (**D**) show 1D slices along the direct ^{13}C dimension through the 2D planes (**A**), (**B**), respectively, at the indicated positions. Source data are provided as a Source Data file.

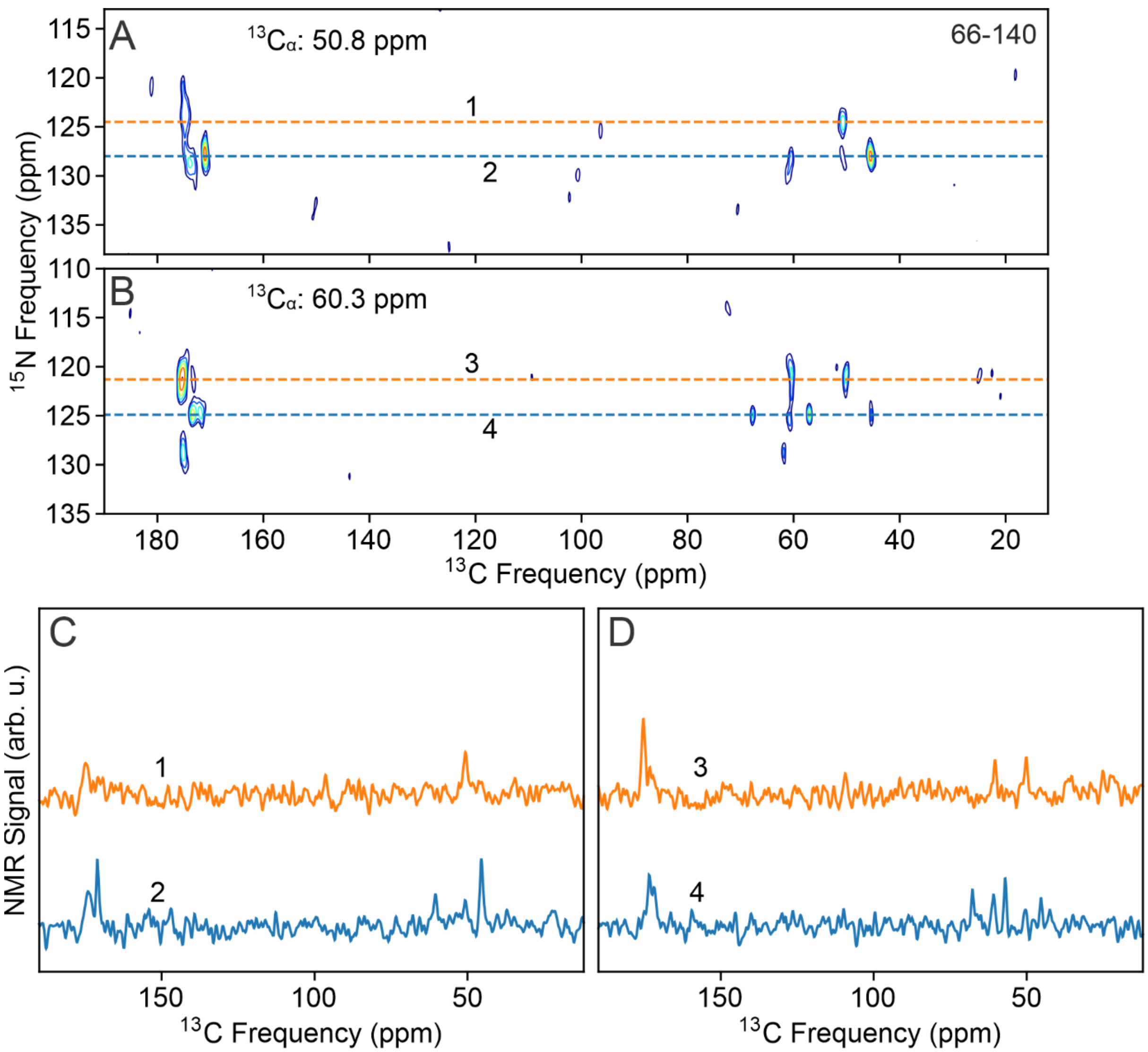

Supplementary Figure 12. 3D CaNCOCX spectrum acquired on ^{13}C,^{15}N-labeled 66-140 fibrils, with 17 kHz MAS. (**A**), (**B**) show representative 2D planes of constant ^{13}Ca frequency acquired in the indirect dimension. Contours are plotted with successive factors of 1.30. 150 Hz Gaussian line broadening was applied in the direct and indirect ^{13}C dimensions, and 120 Hz Gaussian line broadening was applied in the indirect ^{15}N dimension. (**C**), (**D**) show 1D slices along the direct ^{13}C dimension through the 2D planes (**A**), (**B**), respectively, at the indicated positions. Source data are provided as a Source Data file.

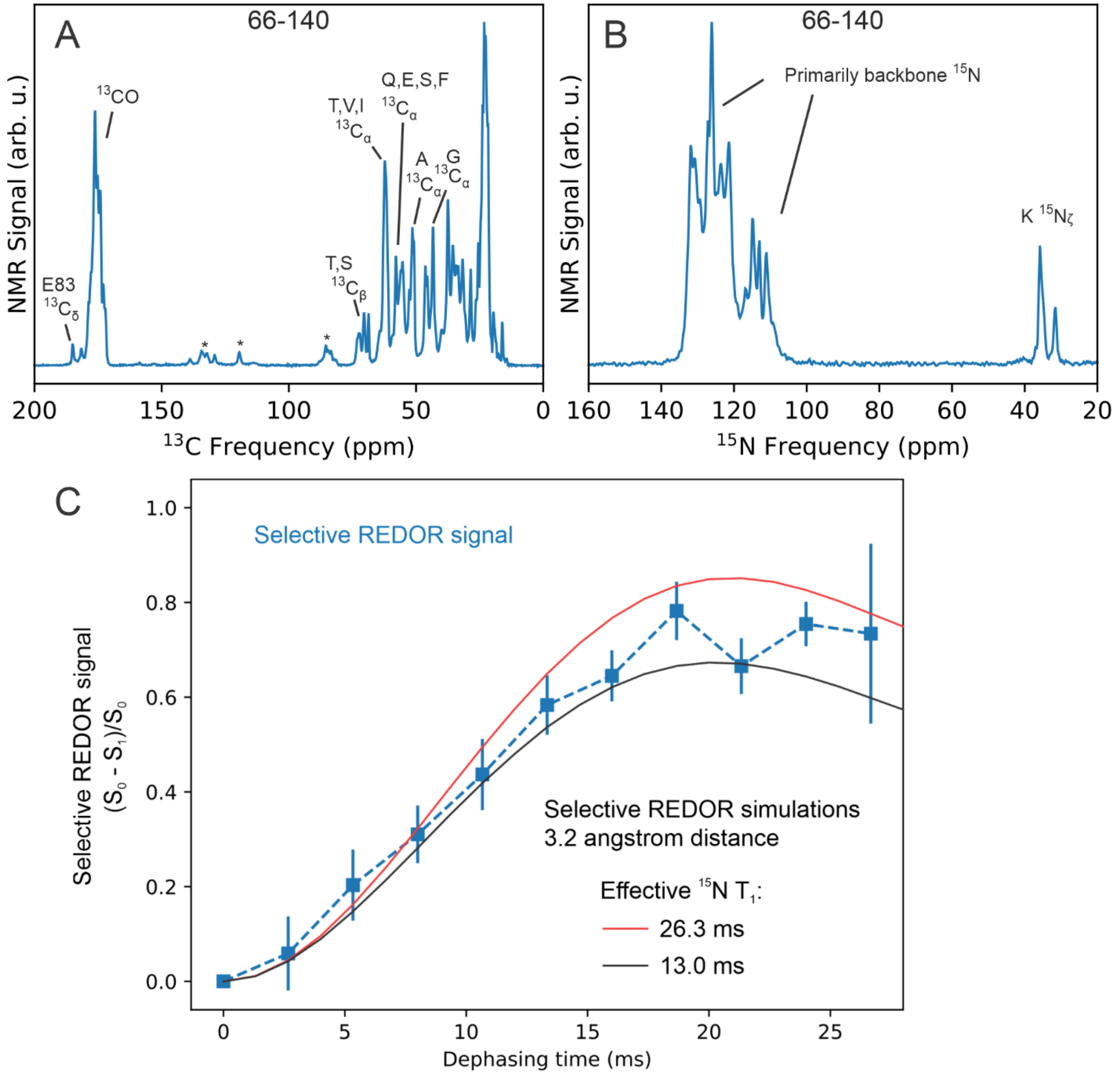

Supplementary Figure 13. (**A**) 1D ^{13}C spectrum acquired on ^{13}C, ^{15}N-labeled 66–140 fibrils with cross polarization (CP) from ^{1}H. Positions of C_α, and C_β signals readily identifiable to a residue type are indicated. The E83 sidechain $^{13}C_\delta$ signal, appearing near 185.0 ppm, is highlighted. (**B**) 1D ^{15}N spectrum acquired on ^{13}C, ^{15}N-labeled 66-140 fibrils with cross polarization (CP) from ^{1}H. Signals falling between 135 ppm and 110 ppm arise from backbone ^{15}N atoms. Signals at between 35 and 30 ppm arise from sidechain ^{15}N atoms in lysine residues. (**C**) Frequency-selective REDOR (FS-REDOR) experiments to quantitatively measure ^{13}C-^{15}N dipolar coupling strength. Spectra were recorded on-resonance with the E83 sidechain $^{13}C_\delta$ signal, at a MAS speed of 12 kHz. A train of two 180° pulses per rotor period was applied to ^{15}N to recouple ^{13}C-^{15}N dipolar coupling during the dephasing time. A selective Gaussian 180° pulse 328 μs long was applied to invert the ^{13}C the signals around 185 ppm, corresponding to the E83 and Q79 sidechain $^{13}C_\delta$ and the backbone ^{13}CO signals. Simultaneously, a selective Gaussian 180° pulse 328 μs long was applied to invert the ^{15}N spins around 35 ppm, corresponding to lysine sidechain ^{15}N. Signals were recorded as a function of the dephasing time either with (S_1) or without (S_0) the selective Gaussian 180° ^{15}N pulse. The fractional difference signal $(S_0 - S_1)/S_0$ shows the amount of dephasing due

to recoupled ^{13}C-^{15}N dipolar coupling between E83 sidechain $^{13}C_{\delta}$ and lysine sidechain ^{15}N. REDOR simulations were carried out assuming a 3.2 angstrom distance with an effective ^{15}N T_1 of 13.0 ms, and of 26.3 ms. Source data are provided as a Source Data file.

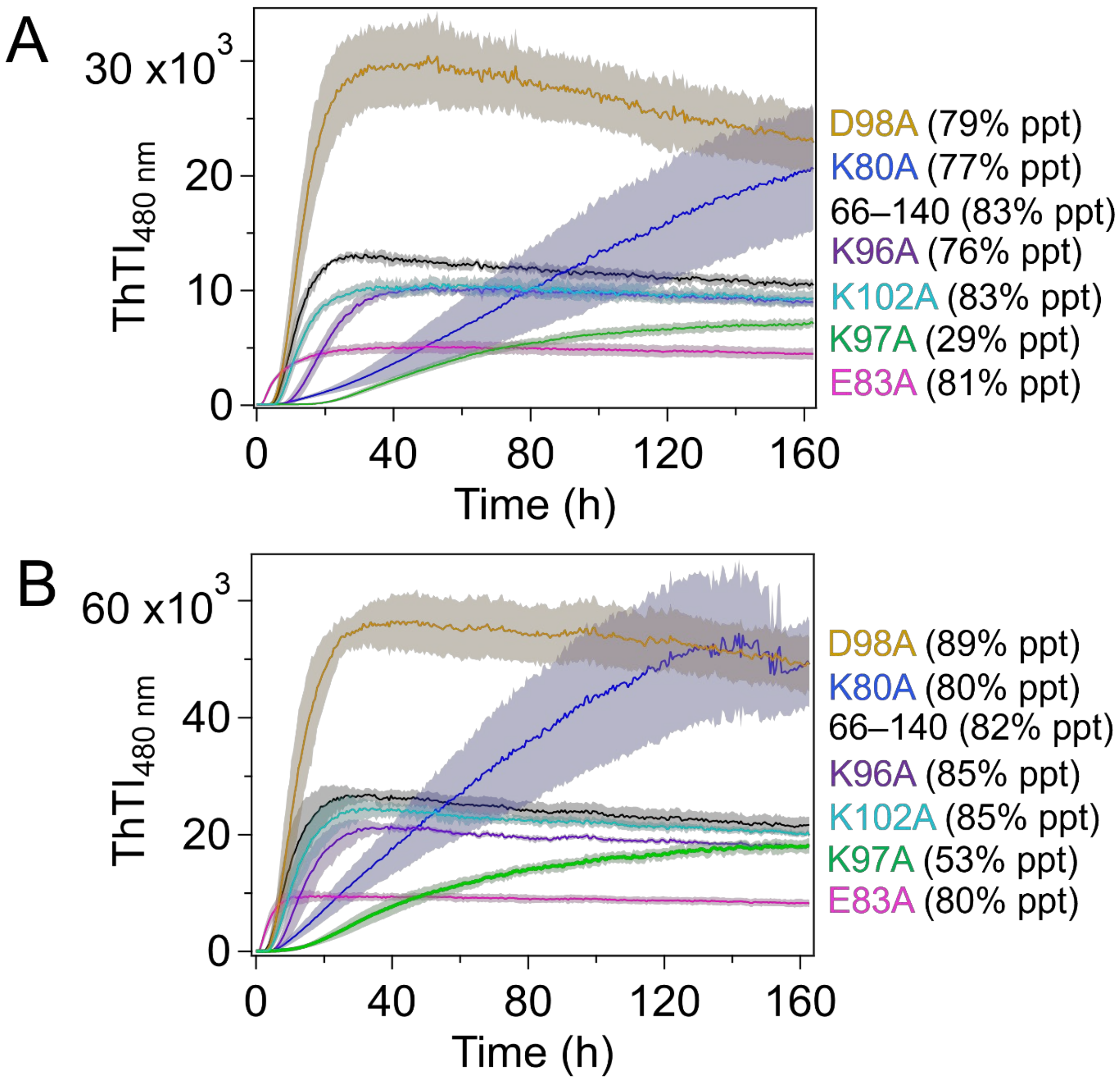

Supplementary Figure 14. Aggregation kinetics monitored by ThT fluorescence of K80A (*blue*), E83A (*magenta*), K96A (*purple*), K97A (*green*), D98A (*gold*) and K102A (*cyan*) protein 40 μM (**A**) and 80 μM (**B**)) in pH 7.4 buffer (20 mM NaPi, 140 mM NaCl) at 37 °C with continuous linear shaking supplemented with a 2-mm glass bead. Solid lines and shaded regions represent mean and SD, respectively ($n \geq 7$). The first 50 h are shown in Fig. 3B. *ppt* refers to amount of insoluble material pelleted by ultracentrifugation. Source data are provided as a Source Data file.

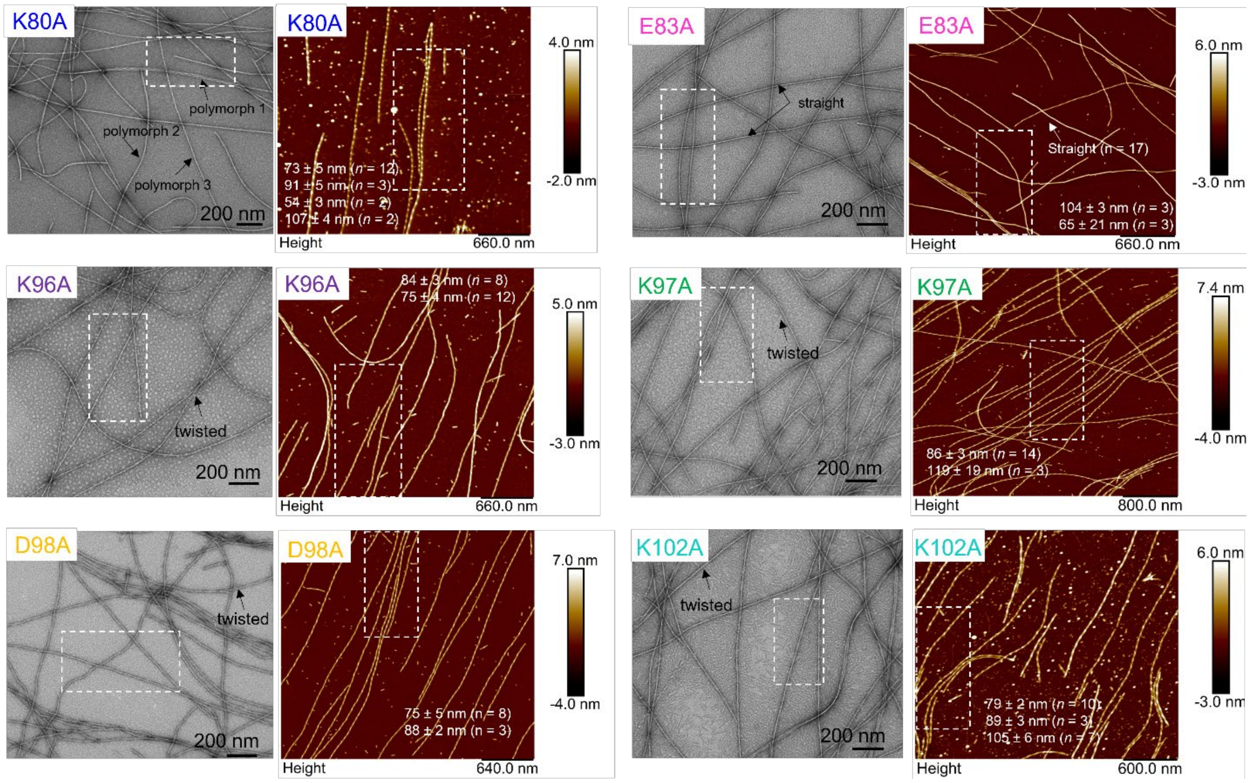

Supplementary Figure 15. Representative TEM (*n* ≥ 6) and AFM (*n* = 5) images of K80A, E83A, K96A, K97A, D98A and K102A fibrils. Dashed areas represent images used in Fig. 4C. Scale bars are as shown. Various helical pitches are reported for each mutant from analyzing between 11 to 23 fibrils by AFM. Due to the low fibril numbers reported from AFM, helical pitches derived from analyzing TEM images (*n* > 50 fibrils) were used to establish the main polymorph for each sample. These data revealed the 73 ± 5 nm for K80A, 84 ± 3 nm for K96A, 86 ± 3 nm for K97A, 88 ± 2 nm for D98A and 89 ± 3 nm for K102A are the main polymorph.

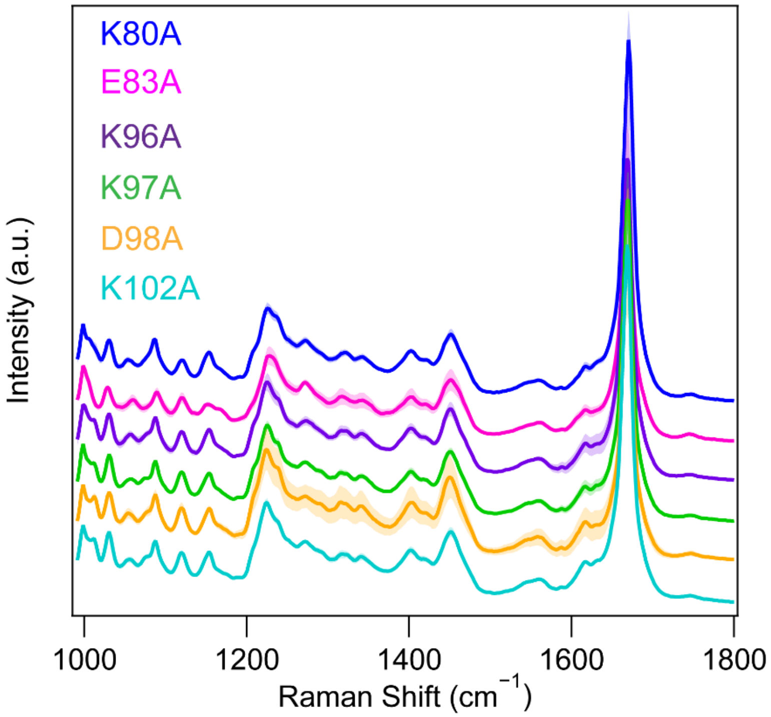

Supplementary Figure 16. Full Raman spectra of single-Ala mutants shown in Fig. 5A. Solid lines and sharded regions represent mean and SD, respectively ($n \geq 15$). Source data are provided as a Source Data file.

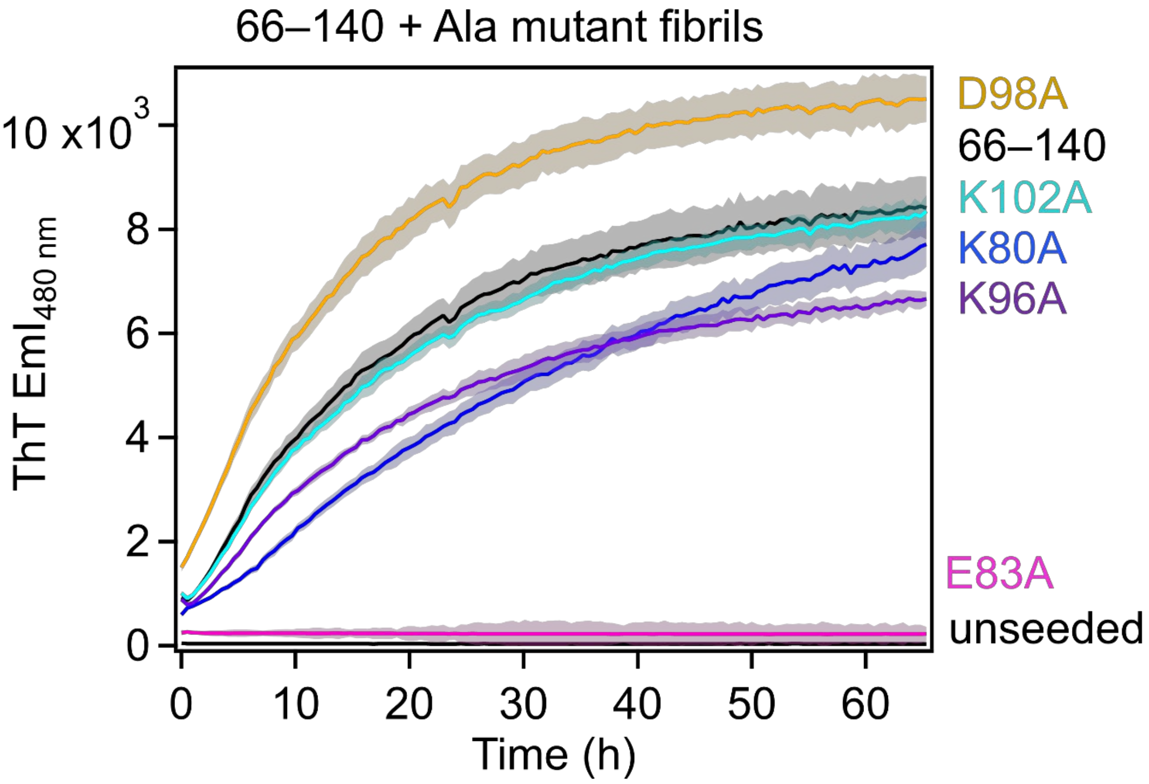

Supplementary Figure 17. Aggregation kinetics monitored by ThT (10 μM) of soluble 66–140 (30 μM) seeded with 1.5 μM 66–140 (*black*), K80A (*blue*), E83A (*magenta*), K96A (*purple*), D98A (*gold*) and K102A (*cyan*) fibrils in pH 7.4 buffer (20 mM NaPi, 140 mM NaCl) at 37 °C with continuous linear shaking. Solid lines and sharded regions represent mean and SD, respectively ($n \geq 6$). Unseeded 66–140 is shown as a control. Source data are provided as a Source Data file.

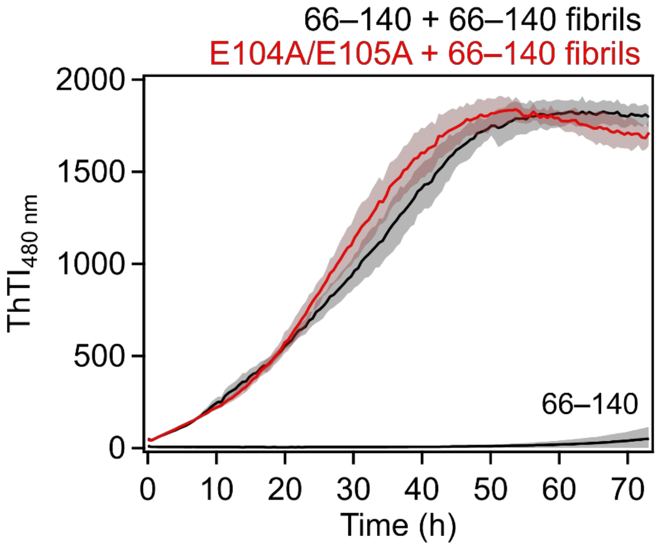

Supplementary Figure 18. Aggregation kinetics monitored by ThT (10 μM) of E104/E105A (red) and 66–140 (black) (30 μM) seeded with 66–140 fibrils (0.3 μM) in pH 7.4 buffer (20 mM NaPi, 140 mM NaCl) at 37 °C with continuous linear shaking. Solid lines and shaded regions represent mean and SD, respectively ($n \geq 6$).Unseeded 66–140 is shown as a control. Source data are provided as a Source Data file.

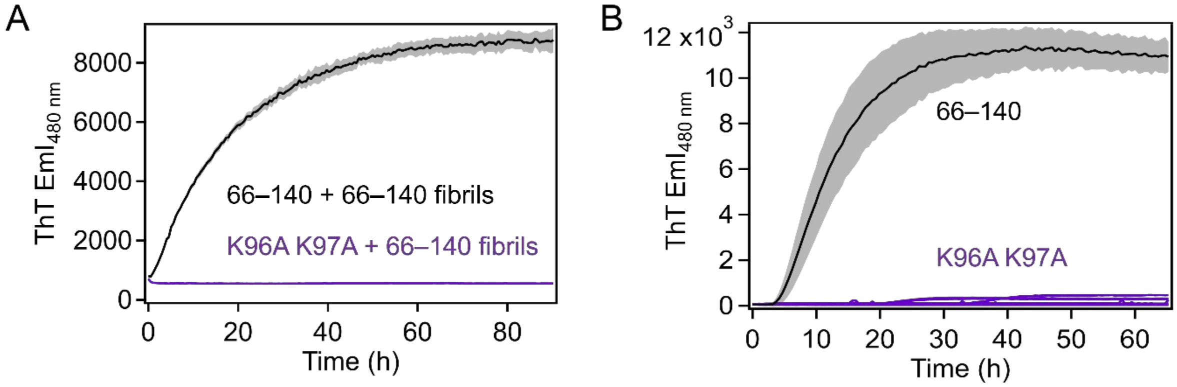

Supplementary Figure 19. Aggregation kinetics monitored by ThT (10 μM) of (**A**) K96A/K97A (purple) and 66–140 (black) (30 μM) seeded with 66–140 fibrils (1.5 μM) in pH 7.4 buffer (20 mM NaPi, 140 mM NaCl) at 37 °C with continuous linear shaking. Solid lines and sharded regions represent mean and SD, respectively ($n \geq 5$). (**B**) K96A/K97A (purple) and 66–140 (black) (40 μM) in pH 7.4 buffer (20 mM NaPi, 140 mM NaCl) at 37 °C with continuous linear shaking supplemented with a 2-mm glass bead. Solid lines and sharded regions represent mean and SD, respectively ($n \geq 12$). Source data are provided as a Source Data file.

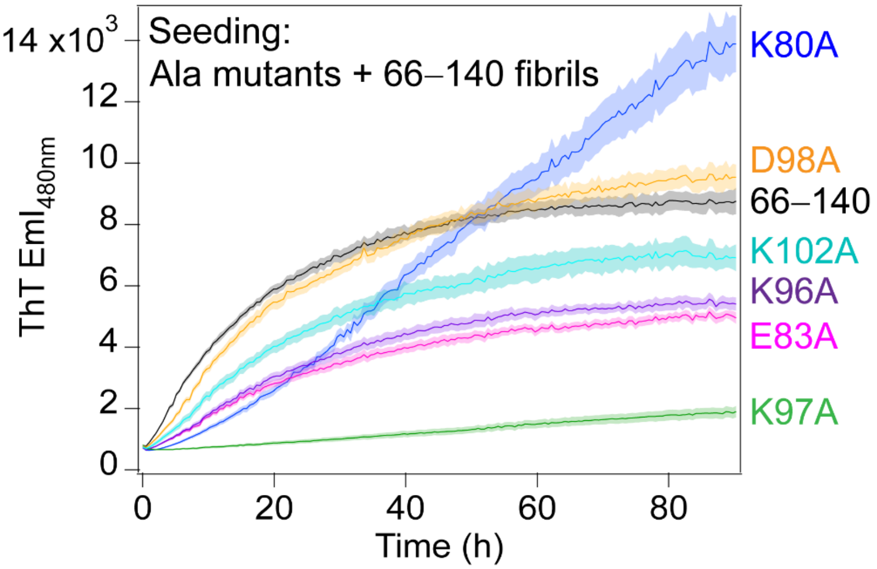

Supplementary Figure 20. Aggregation kinetics monitored by ThT (10 μM) of soluble 66–140 (*black*), K80A (*blue*), E83A (*magenta*), K96A (*purple*), K97A (*green*), D98A (*gold*) and K102A (*cyan*) (30 μM) seeded with 66–140 fibrils (1.5 μM) in pH 7.4 buffer (20 mM NaPi, 140 mM NaCl) at 37 °C with continuous linear shaking. Solid lines and sharded regions represent mean and SD, respectively ($n \geq 5$). Source data are provided as a Source Data file.

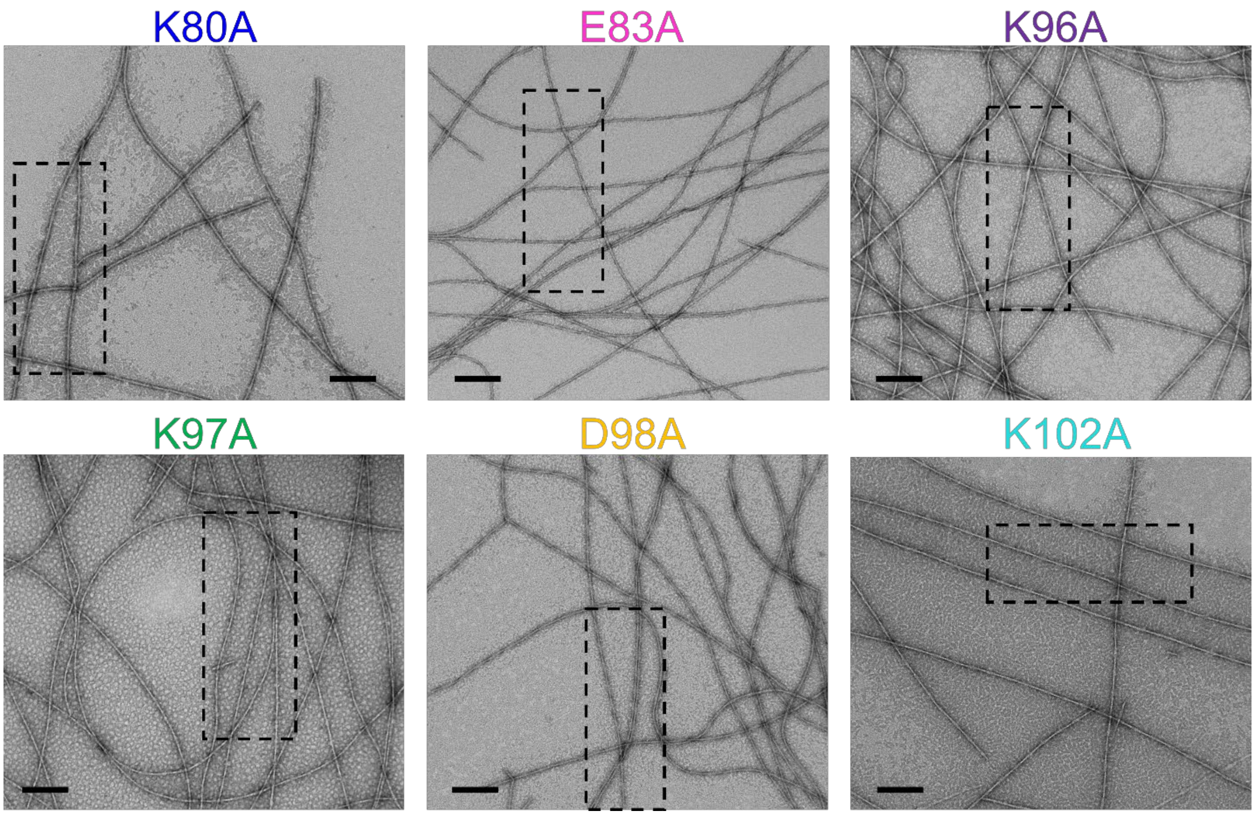

Supplementary Figure 21. Representative TEM images of K80A (*n* = 15), E83A (*n* = 14), K96A (*n* = 14), K97A (*n* = 11), D98A (*n* = 17) and K102A (*n* = 12) (30 μM) cross-seeded with 0.3 μM 66–140 fibrils. Dashed areas represent images shown in Fig. 6B. Scale bars are 200 nm.

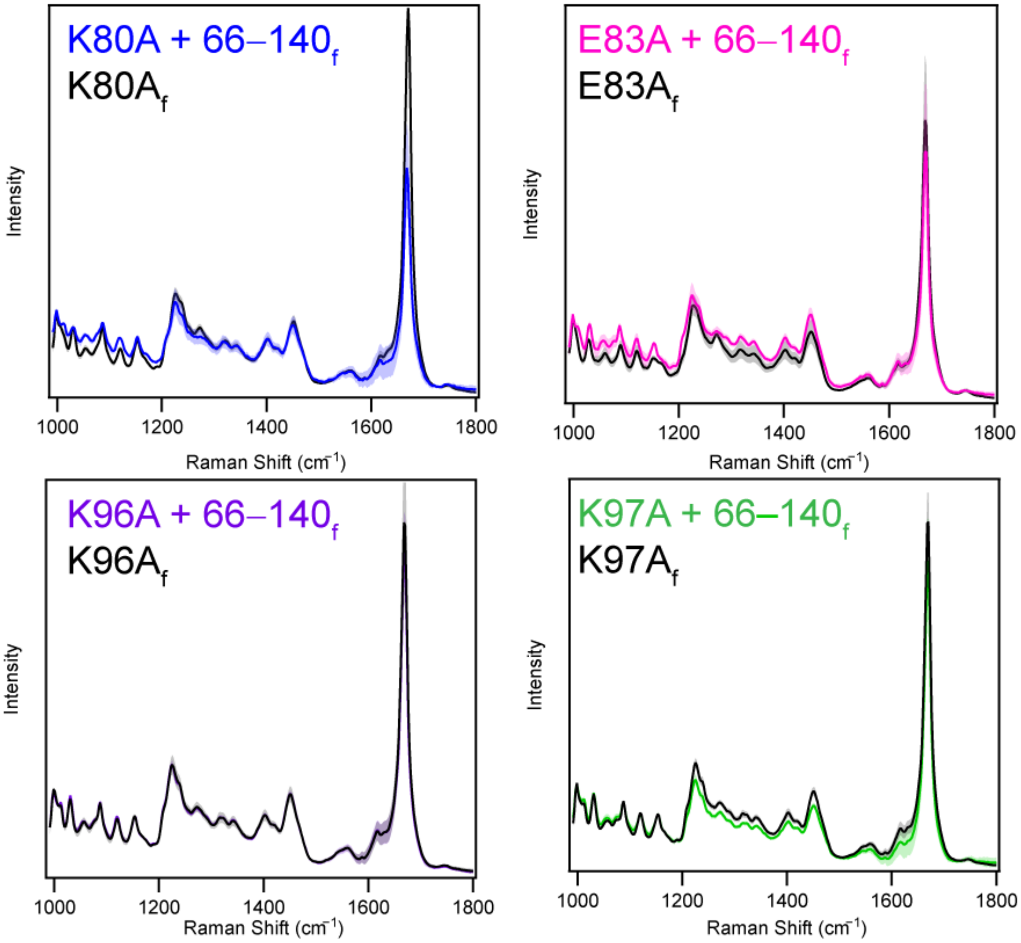

Supplementary Figure 22. Full Raman spectra of cross-seeded single-Ala mutants shown in Fig. 6C. Solid lines and sharded regions represent mean and SD, respectively ($n \geq 15$). Source data are provided as a Source Data file.

E83K/K97E + 66–140 fibrils

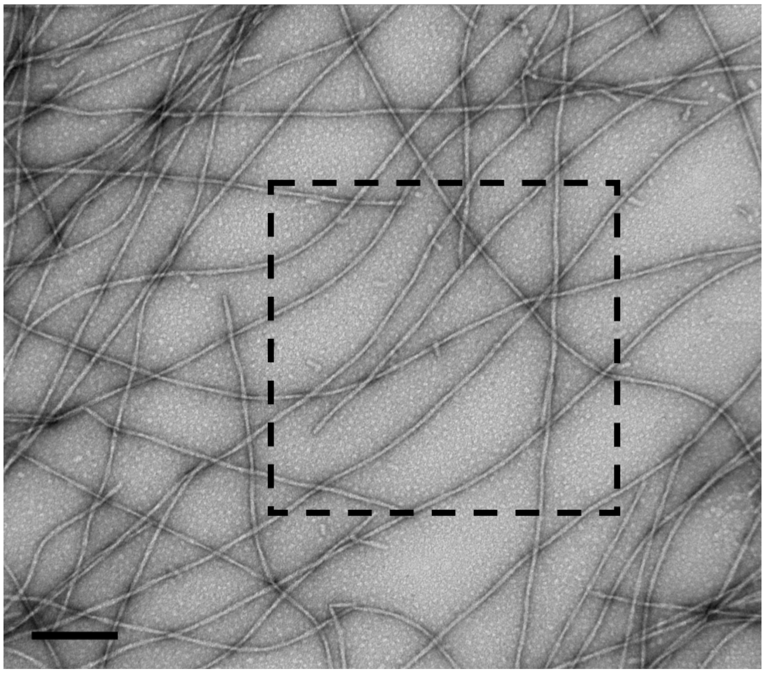

Supplementary Figure 23. Representative TEM image of E83K/K97E (30 µM) seeded with 1.5 µM 66–140 fibrils (*n* = 17). Dashed areas represent images used in Fig. 7B. Scale bar is 200 nm.

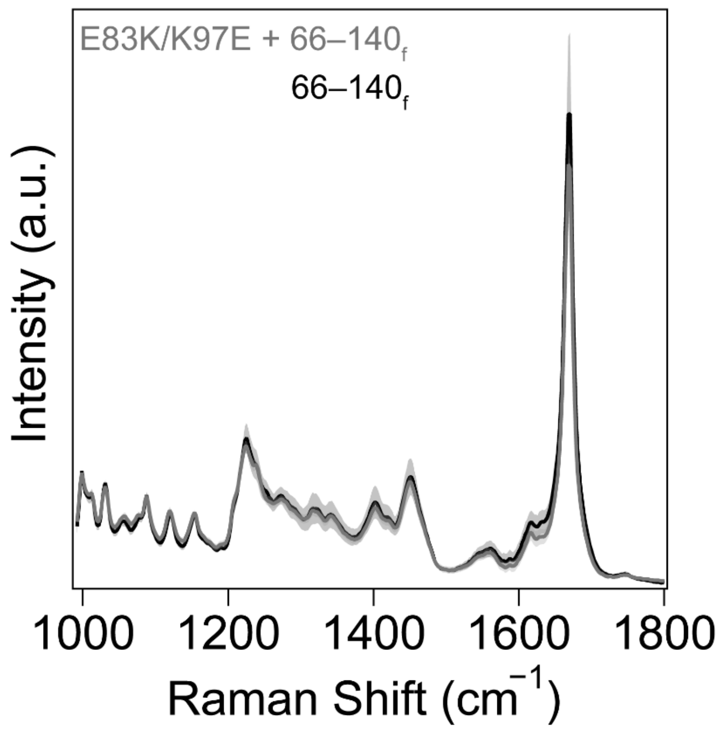

Supplementary Figure 24. Full Raman spectra of cross-seeded E83K/K97E with 66–140 fibrils shown in Fig. 7C. Solid lines and shaded regions represent mean and SD, respectively ($n \geq 15$). Source data are provided as a Source Data file.

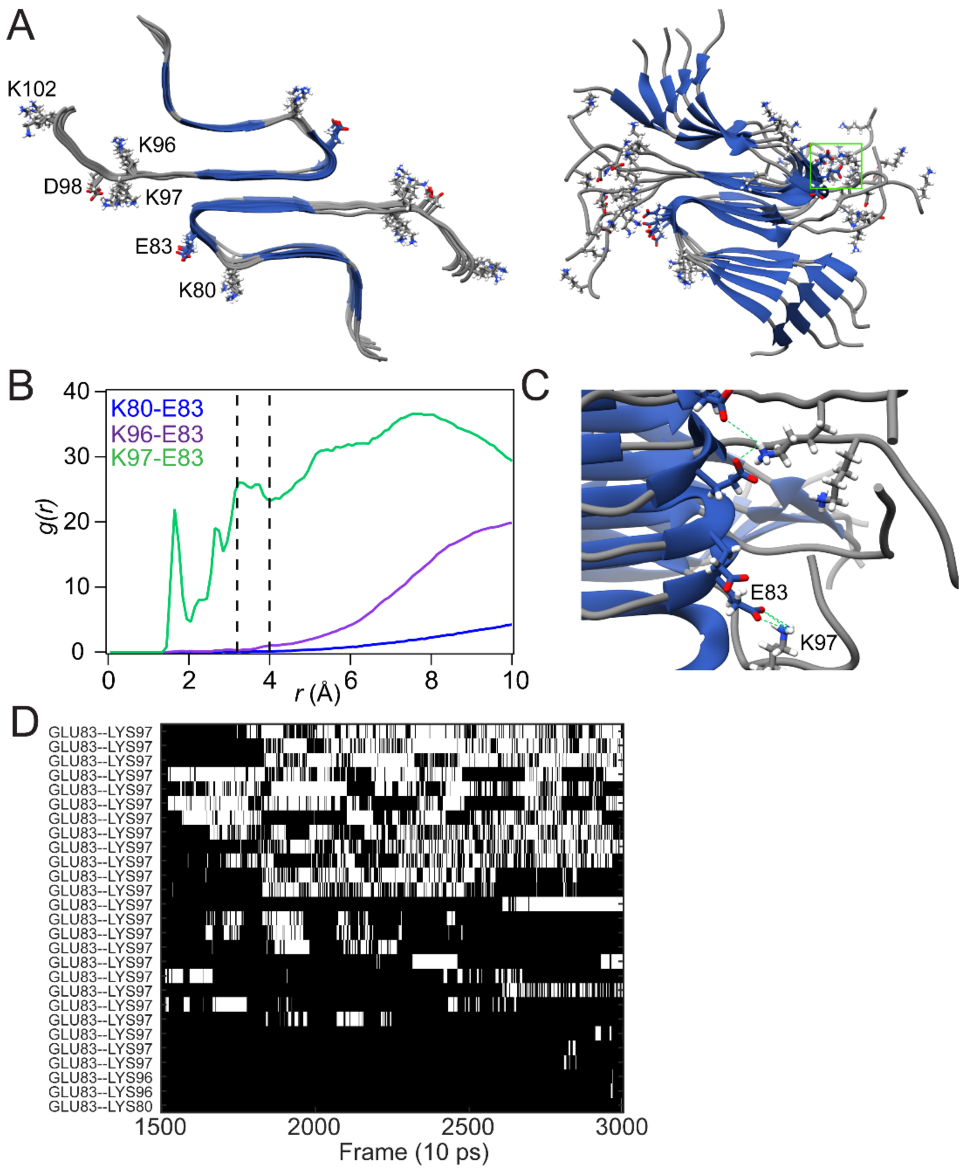

Supplementary Figure 25. 66–140 model structure by molecular dynamic simulations. (**A**) Starting structure (left) and ending structure (right) from a 30-ns MD simulation. (**B**) Radial distribution functions for the distance of K80 (blue), K96 (purple), and K97 (green) to E83. (**C**) A zoomed-in view of possible salt bridges (green, dotted line) between K97 and E83 in the structure depicted in panel *A*, right (green box). (**D**) Salt bridges occurring over the 15-ns MD simulation trajectory analyzed, where white indicates a salt bridge was detected. Top to bottom is ranked by the number of salt bridges. Source data are provided as a Source Data file.

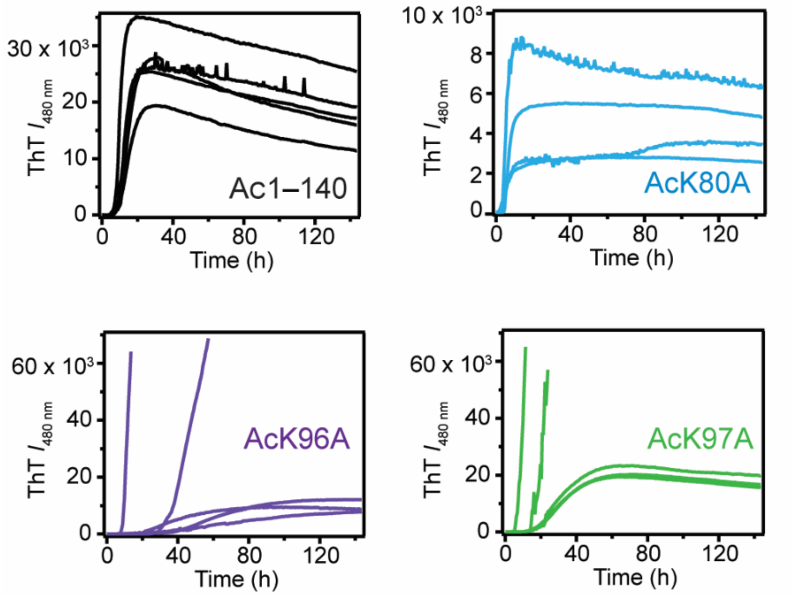

Supplementary Figure 26. Aggregation kinetics monitored by ThT fluorescence of Ac1–140 (*black*), K80A (*cyan*), E83A (*magenta*), K96A (*purple*) and K97A (*green*), protein (100 μM) in pH 7.4 buffer (20 mM NaPi, 140 mM NaCl) at 37 °C with continuous linear shaking supplemented with a 2-mm glass bead ($n \geq 5$). It is noted that two wells in each AcK96A and AcK97A exhibited saturating ThT intensities. Source data are provided as a Source Data file.

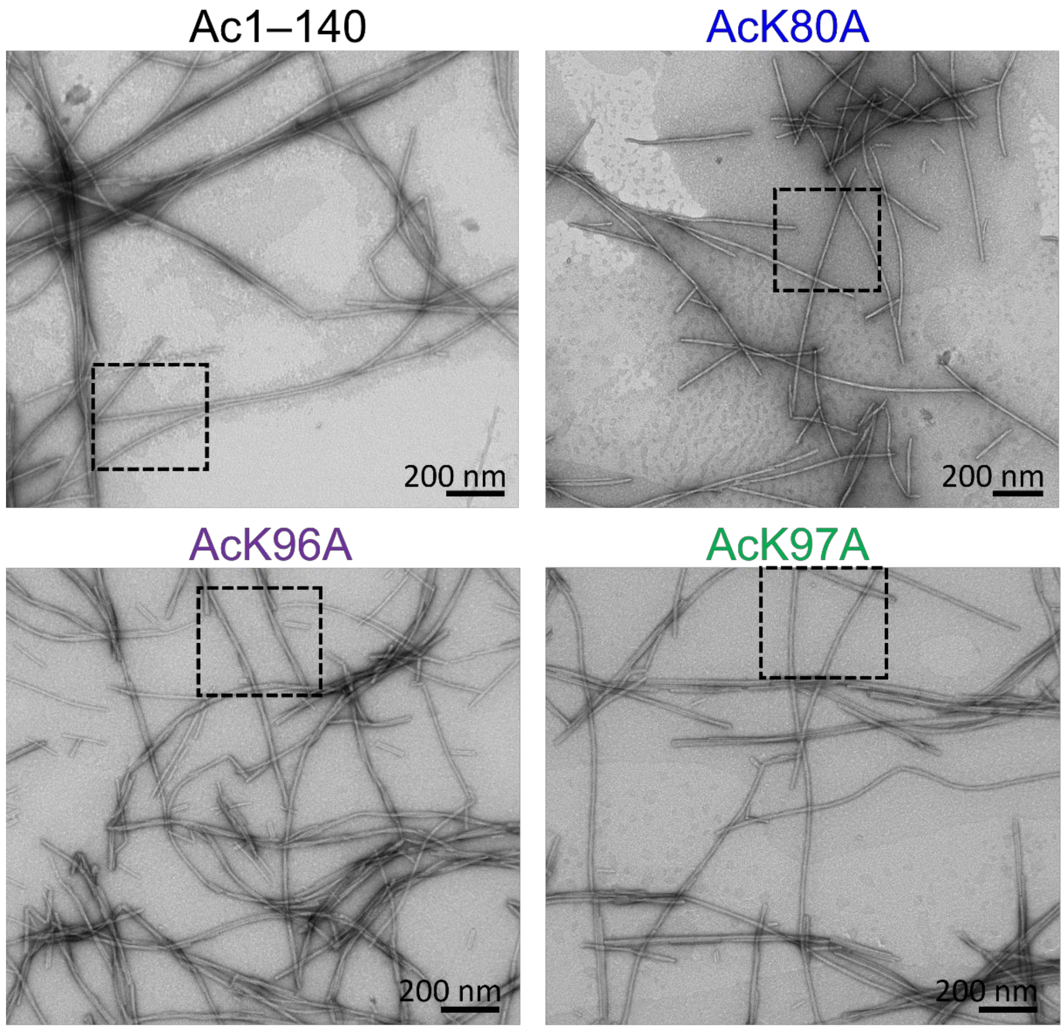

Supplementary Figure 27. Representative TEM images of Ac1–140 (*n* = 11), AcK80A (*n* = 3), AcK96A (*n* = 17), and AcK97A (*n* = 3) fibrils. Dashed areas represent images used in Fig. 8F. Scale bars are as shown.

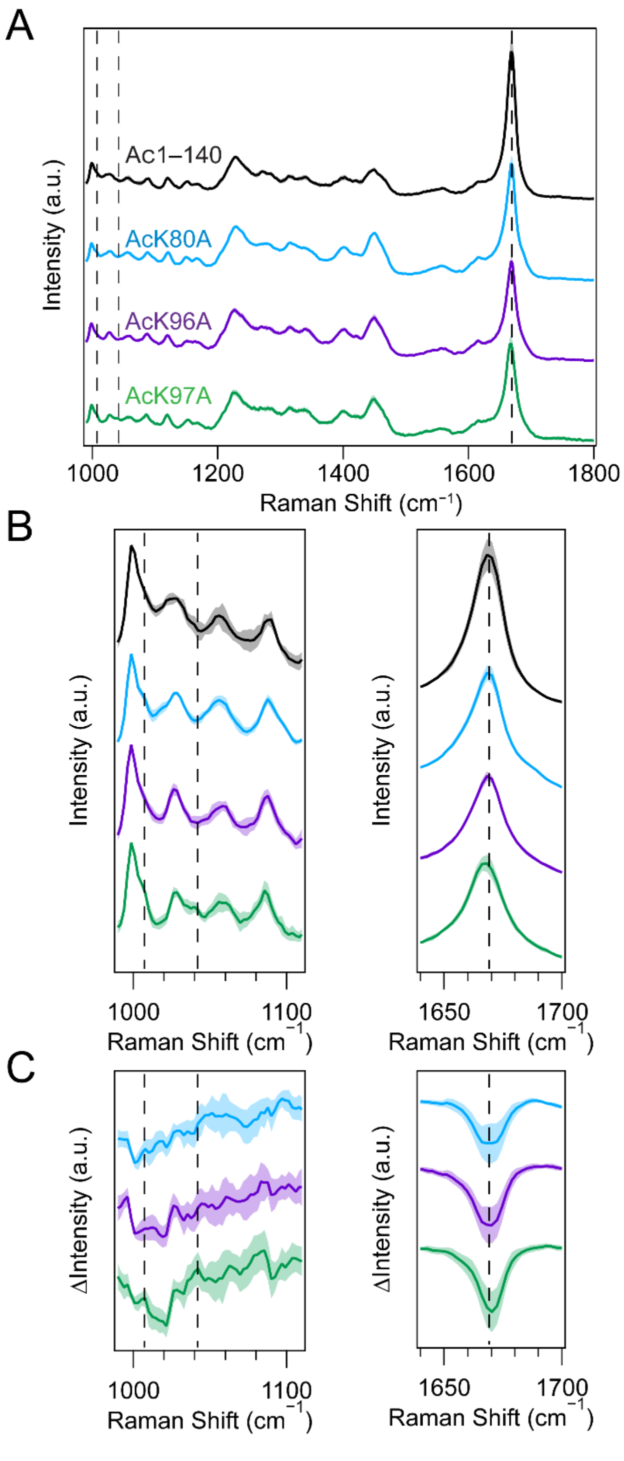

Supplementary Figure 28. (**A**). Full Raman spectra of Ac1-140 and single Ala mutants. Solid lines and shaded regions represent mean and SD, respectively ($n \geq 5$). Dashed lines represent areas of interest. (**B**) Expanded view of the fingerprint (left) and Amide I (right) regions from the spectra in panel A. (**C**) Difference spectra generated by subtracting the average mutant from the average Ac1–140 spectrum. The error has been propagated from the SD of the averaged spectra. Source data are provided as a Source Data file.

Supplementary Table 1. MS analysis of lysosomal digestion of soluble and fibrillar α-syn.

Ac1–140 monomer (15 µM) + lysosomal extract (10 µg)		
Observed Mass (Da)	Theoretical Mass (Da)	Position in α-syn sequence
14502.4	14502.2	Ac1–140
13967.6	13967.6	5–140
13505.9	13506.2	Ac1–132
12937.6	12937.3	15–140
12383.4	12383.0	Ac1–122
10600.1	10599.6	39–140
10346.3	10345.9	Ac1–103
9811.4	9811.3	5–103
9334.9	9334.7	Ac1–94
8958.5	8958.3	Ac1–90
8781.3	8781.0	15–103
8202.6	8202.4	Ac1–81
7757.3	7757.4	66–140
7872.0	7871.5	65–140
7403.6	7403.5	Ac1–73
6763.0	6762.8	Ac1–65
5563.2	5561.9	91–140
5185.7	5185.5	95–140
4174.3	4174.3	104–140

Ac1–140 fibrils (15 µM) + lysosomal extract (30 µg)		
Observed Mass (Da)	Theoretical Mass (Da)	Position in α-syn sequence
14502.8	14502.2	Ac1–140
13967.7	13967.6	5–140
13451.3	13450.9	10–140
13320.2	13320.0	Ac1–130
12937.6	12937.3	15–140
12875.9	12875.5	Ac1–126
12668.4	12668.0	18–140
12583.5	12583.2	Ac1–124
12383.3	12383.0	Ac1–122
12057.5	12056.7	Ac1–119
11848.6	11848.4	5–122
11499.4	11499.0	Ac1–114
11332.7	11331.8	10–122
11144.0	11143.6	Ac1–111
10448.0	10447.8	10–114
10345.9	10345.9	Ac1–103
9934.7	9934.2	15–114
9933.1	9933.4	Ac1–99
9664.9	9664.9	18–114

Supplementary Table 2. MS analysis of individual Cts B, L, D and AEP digestion of soluble α-syn.

Ac1–140 monomer (15 μM) + CtsB (15 nM)

Observed Mass (Da)	Theoretical Mass (Da)	Position in α-syn sequence
14502.7	14502.2	Ac1–140
13651.3	13651.2	8–140
12937.6	12937.3	15–140
12738.9	12739.1	17–140
11854.2	11854.1	26–140
10599.7	10599.6	39–140
9087.2	9086.9	54-140
8958.6	8958.3	Ac1–90
8202.7	8202.4	Ac1–81
7871.6	7871.5	65–140
7601.3	7601.2	68–140
7393.5	7393.4	15–90
6191.5	6191.2	Ac1–60
6448.7	6448.4	Ac1–62
6089.7	6089.5	84–140
5562.0	5561.9	91–140
5534.6	5534.4	Ac1–54
5055.9	5055.7	39–90
5206.5	5206.0	Ac1–50

Ac1–140 monomer (15 μM) + CtsL (15 nM)

Observed Mass (Da)	Theoretical Mass (Da)	Position in α-syn sequence
14502.5	14502.2	Ac1–140
13836.4	13836.4	6–140
13450.5	13450.9	10-140
12668.5	12668.0	18–140
11683.7	11683.9	28–140
11499.3	11499.1	Ac1–114
10599.9	10599.6	39–140
10346.4	10345.9	Ac1–103
10280.5	10280.3	42–140
9314.3	9314.2	51–140
9087.1	9086.9	54–140
7603.7	7603.7	Ac1–75
7601.3	7601.2	68–140
6919.0	6918.9	Ac1–67
6916.6	6916.44	76–140
6675.1	6675.2	79–140
6089.8	6089.4	84–140
5433.6	5433.3	Ac1–53
5206.3	5206.0	Ac1–50
4240.1	4239.9	Ac1–41
4183.1	4182.9	Ac1–40

Ac1–140 monomer (15 μM) + AEP (15 nM)

Observed Mass (Da)	Theoretical Mass (Da)	Position in α-syn sequence
14502.6	14502.2	Ac1–140
12383.5	12383.0	Ac1–122
10346.3	10345.9	Ac1–103
7757.6	7757.41	66–140
6763.0	6762.8	Ac1–65
4173.7	4174.3	104–140

Ac1–140 monomer (15 μM) + CtsD (15 nM)

Observed Mass (Da)	Theoretical Mass (Da)	Position in α-syn sequence
14502.5	14502.2	Ac1–140
13967.9	13967.6	5–140
13506.5	13506.2	Ac1–132
12583.5	12583.2	Ac1–124
12048.7	12048.6	5–124
11745.7	11745.4	Ac1–116
11210.9	11210.8	5–116
9334.9	9334.7	Ac1–94

Supplementary Table 3. MS analysis of Cts B, L, D and AEP digestion of α-syn fibrils.

Ac1–140 fibrils (15 μM) + CtsB (300 nM)

Observed Mass (Da)	Theoretical Mass (Da)	Position in α-syn sequence
14501.8	14502.2	Ac1–140
12383.3	12383.0	Ac1–122
11087.0	11086.6	Ac1–110
10958.0	10957.5	Ac1–109
10818.5	10818.2	15–122
10661.5	10661.2	Ac1–106
10475.2	10475.0	Ac1–104
9933.6	9933.4	Ac1–99
9792.2	9792.0	25–122
9735.1	9734.9	26–122
9521.9	9521.8	15–110
9392.7	9392.6	15–109
8368.2	8368.5	15–99
7342.4	7342.3	25–99
7285.4	7285.3	26–99

Ac1–140 fibrils (15 μM) + CtsL (300 nM)

Observed Mass (Da)	Theoretical Mass (Da)	Position in α-syn sequence
14501.4	14502.2	Ac1–140
13320.4	13320.0	Ac1–130
12937.7	12937.3	15–140
12739.3	12739.1	17–140
12397.6	12396.7	21–140
12383.4	12383.0	Ac1–122
11755.3	11755.2	15–130
11499.5	11499.0	Ac1–114
11086.7	11086.6	Ac1–110
10958.2	10957.5	Ac1–109
10818.5	10818.2	15–122
10548.9	10548.8	18–122
10346.2	10345.9	Ac1–103
10277.8	10277.5	21–122
9934.3	9934.2	15–114
9521.8	9521.8	15–110
9664.7	9664.9	18–114
9393.0	9392.6	15–109

Ac1–140 fibrils (15 μM) + AEP (300 nM)

Observed Mass (Da)	Theoretical Mass (Da)	Position in α-syn sequence
14502.6	14502.2	Ac1–140
14214.6	14213.9	3–140
13912.6	13912.6	Ac1–135
12383.5	12383.0	Ac1–122
12056.7	12056.7	Ac1–119
12095.0	12094.7	3–122
11614.4	11614.2	Ac1–115
11326.3	11325.9	3–115
11087.1	11086.6	Ac1–110
10798.6	10798.3	3–110
10346.3	10345.9	Ac1–103
10057.9	10057.6	3–103

Ac1–140 fibrils (15 μM) + CtsD (300 nM)

Observed Mass (Da)	Theoretical Mass (Da)	Position in α-syn sequence
14502.6	14502.2	Ac1–140
13968.4	13967.6	5–140
12582.5	12583.2	Ac1–124
11745.8	11745.4	Ac1–116
11499.5	11499.1	Ac1–114
11144.0	11143.6	Ac1–111
12049.0	12048.6	5–124
11211.1	11210.8	5–116
10964.5	10964.5	5–114
10609.4	10609.1	5–111

Supplementary Table 4. Summary of NMR measurement conditions. MAS = magic angle spinning frequency, Na = number of scans per free induction decay, T_{rep}= delay between scans, t_{1max} = maximum t_1 value used, t_{1inc} = t_1 increment, t_{2max} = maximum t_1 value used, t_{2inc} = t_2 increment, τ_{PDSD} = DARR/RAD mixing time, τ_{XY} = cross-polarization period, where X and Y are the two nuclei involved (H/^{1}H, C/^{13}C, N/^{15}N).

Spectrum	NMR Parameters	Total Time
2D CC	MAS = 17 kHz, Na = 64, T_{rep} = 2 s, t_{1max} = 5.12 ms , t_{1inc} = 20 μs, τ_{PDSD} = 25 ms, τ_{XY} = 1.5 ms	18.2 hours
2D NCACX	MAS = 17 kHz, Na = 256, T_{rep} = 2 s, t_{1max} = 9.52 ms, t_{1inc} = 70 μs, τ_{PDSD} = 25 ms, τ_{HN} = 1.5 ms, τ_{NC} = 4.5 ms	38.7 hours
2D NCOCX	MAS = 17 kHz, Na = 256, T_{rep} = 2 s, t_{1max} = 9.52 ms, t_{1inc} = 70 μs, τ_{PDSD} = 25 ms, τ_{HN} = 1.5 ms, τ_{NC} = 4.5 ms	38.7 hours
3D NCACX	MAS = 17 kHz, Na = 32, T_{rep} = 1.5 s, t_{1max} = 6.08 ms, t_{1inc} = 160 μs, t_{2max} = 3.52 ms, t_{2inc} = 80 μs, τ_{PDSD} = 25 ms, τ_{HN} = 1.5 ms, τ_{NC} = 4.5 ms	89.2 hours
3D NCOCX	MAS = 17 kHz, Na = 32, T_{rep} = 1.5 s, t_{1max} = 6.08 ms, t_{1inc} = 160 μs, t_{2max} = 3.52 ms, t_{2inc} = 80 μs, τ_{PDSD} = 25 ms, τ_{HN} = 1.5 ms, τ_{NC} = 4.5 ms	89.2 hours
3D CANCOCX	MAS = 17 kHz, Na = 8x3, T_{rep} = 2 s, t_{1max} = 3.52 ms, t_{1inc} = 80 μs, t_{2max} = 6.0 ms, t_{2inc} = 200 μs, τ_{PDSD} = 35 ms	93.9 hours
FS-REDOR	MAS = 12 kHz, T_{rep} = 2 s, $t_{REDOR,max}$ = 26.67 ms, $t_{redor,inc}$ = 2.67 ms	86.8 hours

Supplementary Table 5. Chemical shift assignments from solid-state NMR spectra of ^{13}C, ^{15}N-labeled 66−140 fibrils. Chemical shifts are in parts per million relative to DSS for ^{13}C and relative to NH_3 for ^{15}N. Uncertainties are ± 0.2 ppm for ^{13}C and ± 0.3 ppm for ^{15}N. Backbone φ and ψ torsion angles were predicted with TALOS-N, using these chemical shifts, with errors estimated by TALOS-N. Predictions marked * were classified as "warn" by TALOS-N. All other predictions are "strong".

Residue	Chemical Shifts (ppm)				Predicted torsion angles (°)	
	15N	13Ca	13CO	13Cb	φ	ψ
V66		60.9	173.4			
G67	112.0	44.5	173.7			
G68	111.9	44.6	171.9			
A69	123.4	51.2	175.2	23.5	-115 +/- 19	138 +/- 11
V70	122.8	61.7	175.2	34.3	-106 +/- 15	129 +/- 9
V71	128.8	61.0	174.8		-102 +/- 11	122 +/- 9
T72	127.1	61.5	172.9	71.1		
G73	113.6	45.2	171.5		-125 +/- 37	155 +/- 22
V74	125.5	61.0	174.2	36.2	-124 +/- 10	128 +/- 5
T75	125.3	61.1	173.1	69.1	-100 +/- 14	127 +/- 5
A76	129.3	50.0	175.3	22.8	-122 +/- 10	132 +/- 9
V77	120.7	60.1	173.5	36.7	-126 +/- 11	131 +/- 7
A78	128.8	50.3	175.2		-88 +/- 15	129 +/- 8
Q79	127.1	54.1	175.4	33.3		
K80	124.9				-82 +/- 9	122 +/- 12
T81	124.7	60.8	174.5	71.8		
V82	125.3	61.4	175.9		-95 +/- 15	126 +/- 9
E83	130.3	55.5	174.0	32.8		
G84	113.9	45.4	171.0		-90 +/- 24	149 +/- 22
A85	128.0	50.5	175.1	24.4		
G86	109.8	44.6	172.0		-147 +/- 44*	165 +/- 21*
S87	122.4	57.0	173.3	67.6	-136 +/- 12	148 +/- 10
I88	124.9	60.3	173.1	42.2	-126 +/- 13	130 +/- 7
A89	130.7	49.8	174.3	22.7	-116 +/- 11	132 +/- 8
A90	126.2	49.7	176.7	22.2	-121 +/- 15	128 +/- 6
A91	129.9	51.5	175.3	22.7	-118 +/- 11	129 +/- 6
T92	120.0	60.9	173.6	69.5	-105 +/- 16	128 +/- 13
G93	111.9	44.3	171.8		-116 +/- 32*	157 +/- 28*
F94	119.8	56.6	175.1	42.2		
V95	123.5	61.2	174.7			

Supplementary Table 6. MS analysis of PK digestion of 66–140 fibrils.

66–140 fibrils (30 μM) + Proteinase K (2 and 0.2 ng total)		
Observed Mass (Da)	Theoretical Mass (Da)	Position in α-syn sequence
7757.69	7757.41	66–140
6001.82	6001.63	66–125
4626.22	4625.21	66–113

Supplementary Table 7. Values of t_{lag} and $t_{½}$ for 66–140 constructs determined by ThT fluorescence. Kinetics are shown in Figs 4B and S14.

	80 µM protein		40 µM protein	
	t_{lag} (h)	$t_{1/2}$ (h)	t_{lag} (h)	$t_{1/2}$ (h)
66-140	2.2 ± 0.3	10.6 ± 2.6	4.1 ± 0.3	11.9 ± 1.4
K80A	3.3 ± 0.8	N.D.	5.5 ± 0.7	N.D.
E83A	1.2 ± 0.2	3.7 ± 0.2	1.1 ± 0.0	6.5 ± 0.8
K96A	4.4 ± 0.2	14.7 ± 2.9	7.9 ± 0.5	20.3 ± 1.9
K97A	2.3 ± 0.7	48.2 ± 6.4	13.5 ± 1.0	55.1 ± 3.4
D98A	2.6 ± 0.7	13.5 ± 3.6	3.8 ± 0.4	13.7 ± 1.8
K102A	3.1 ± 0.4	12.1 ± 1.8	5.5 ± 0.2	13.1 ± 1.4

Supplementary Table 8. MS analysis of PK digestion of single-Ala mutant fibrils.

K80A fibrils (30 μM) + Proteinase K (2 and 0.2 ng total)

Observed Mass (Da)	Theoretical Mass (Da)	Position in α-syn sequence
7700.61	7700.32	66–140
5944.77	5944.53	66–125
4568.30	4568.12	66–113
4154.90	4155.63	66–109

E83A fibrils (30 μM) + Proteinase K (2 and 0.2 ng total)

Observed Mass (Da)	Theoretical Mass (Da)	Position in α-syn sequence
7699.61	7699.37	66–140
5943.88	5943.59	66–125
4154.85	4567.18	66–109

K96A fibrils (30 μM) + Proteinase K (2 and 0.2 ng total)

Observed Mass (Da)	Theoretical Mass (Da)	Position in α-syn sequence
7700.58	7700.32	66–140
5944.76	5944.53	66–125
4568.10	4568.12	66–113
4154.85	4155.63	66–109

K97A fibrils (30 μM) + Proteinase K (2 and 0.2 ng total)

Observed Mass (Da)	Theoretical Mass (Da)	Position in α-syn sequence
7700.60	7700.32	66–140
5944.73	5944.53	66–125
4568.20	4568.12	66–113

D98A fibrils (30 μM) + Proteinase K (2 and 0.2 ng total)

Observed Mass (Da)	Theoretical Mass (Da)	Position in α-syn sequence
7713.68	7713.40	66–140
5957.92	5957.62	66–125
4581.4	4581.20	66–113

K102A fibrils (30 μM) + Proteinase K (2 and 0.2 ng total)

Observed Mass (Da)	Theoretical Mass (Da)	Position in α-syn sequence
7700.59	7700.32	66–140
5944.76	5944.53	66–125
4568.10	4568.12	66–113
