# Supplementary figures and images for: Defining essential charged residues in fibril formation of a lysosomal derived N-terminal α-synuclein truncation
Source: Nat Commun. 2025 Apr 23;16:3825. doi: 10.1038/s41467-025-58899-9 (PMC12019160; doi:10.1038/s41467-025-58899-9)

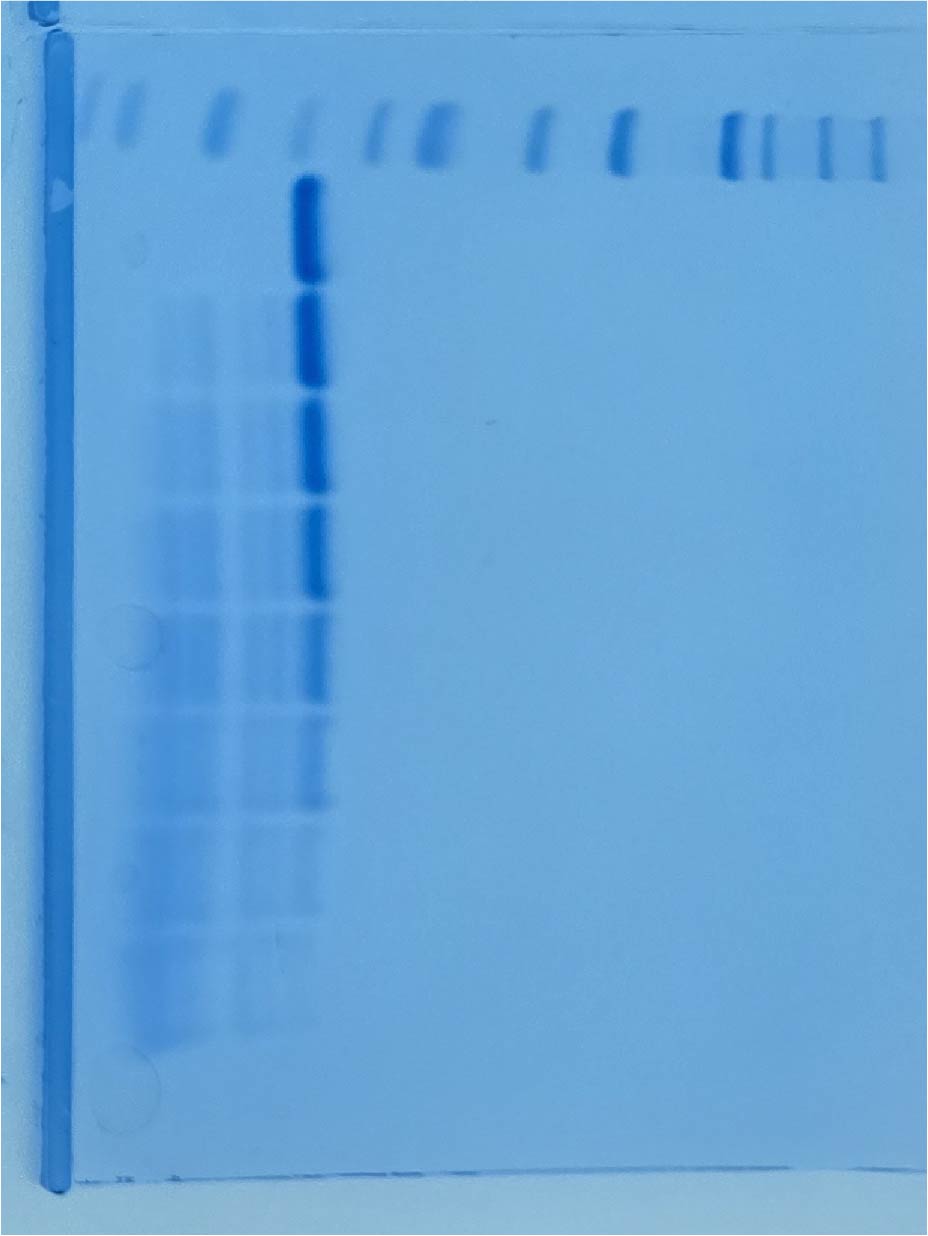

Supplement: Supplementary file 4 — Source Data [file 41467_2025_58899_MOESM4_ESM.zip › Full gel of Supplementary Figure 1a.jpg]

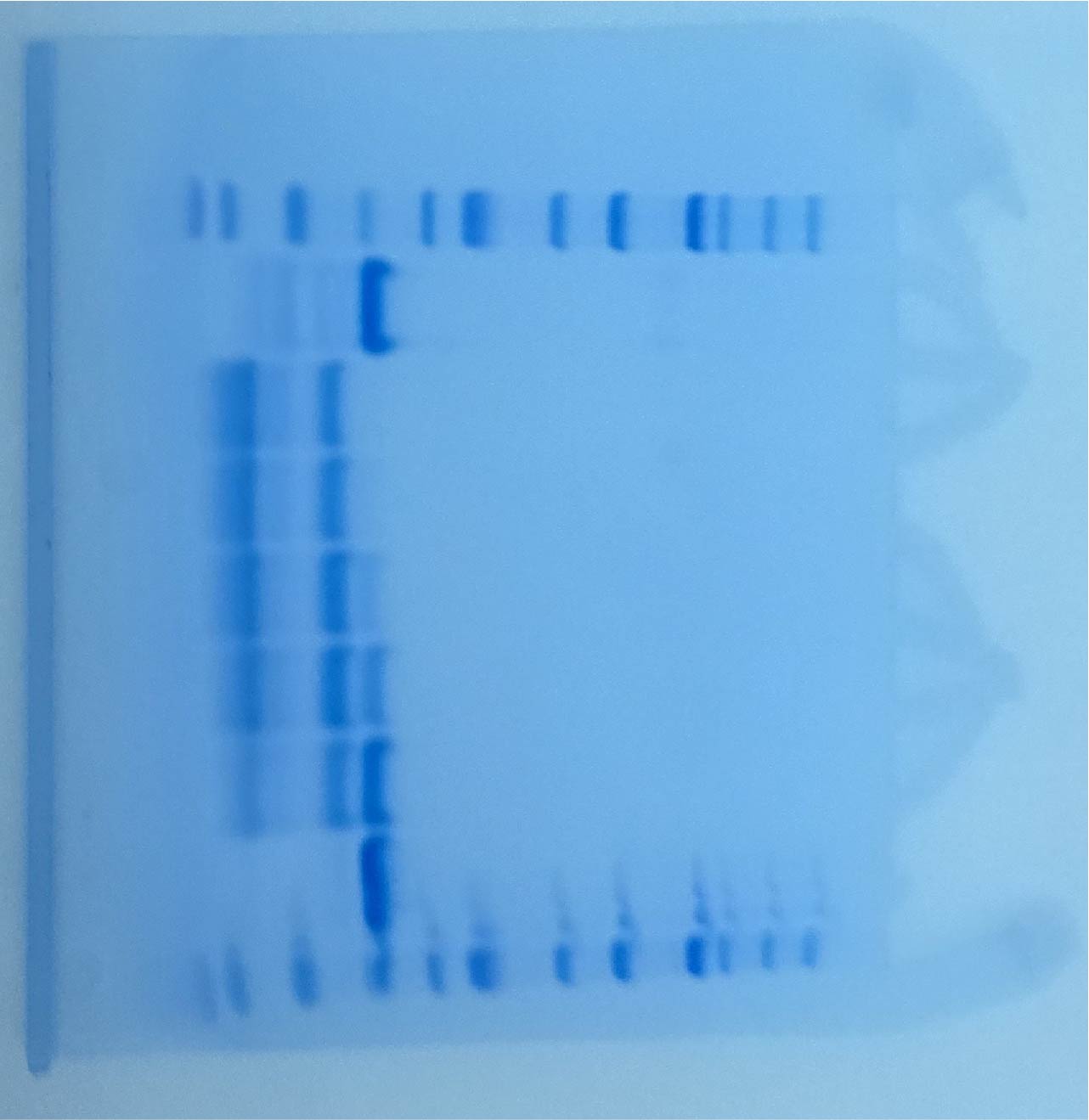

Supplement: Supplementary file 4 — Source Data [file 41467_2025_58899_MOESM4_ESM.zip › Full gel of Supplementary Figure 1b.jpg]

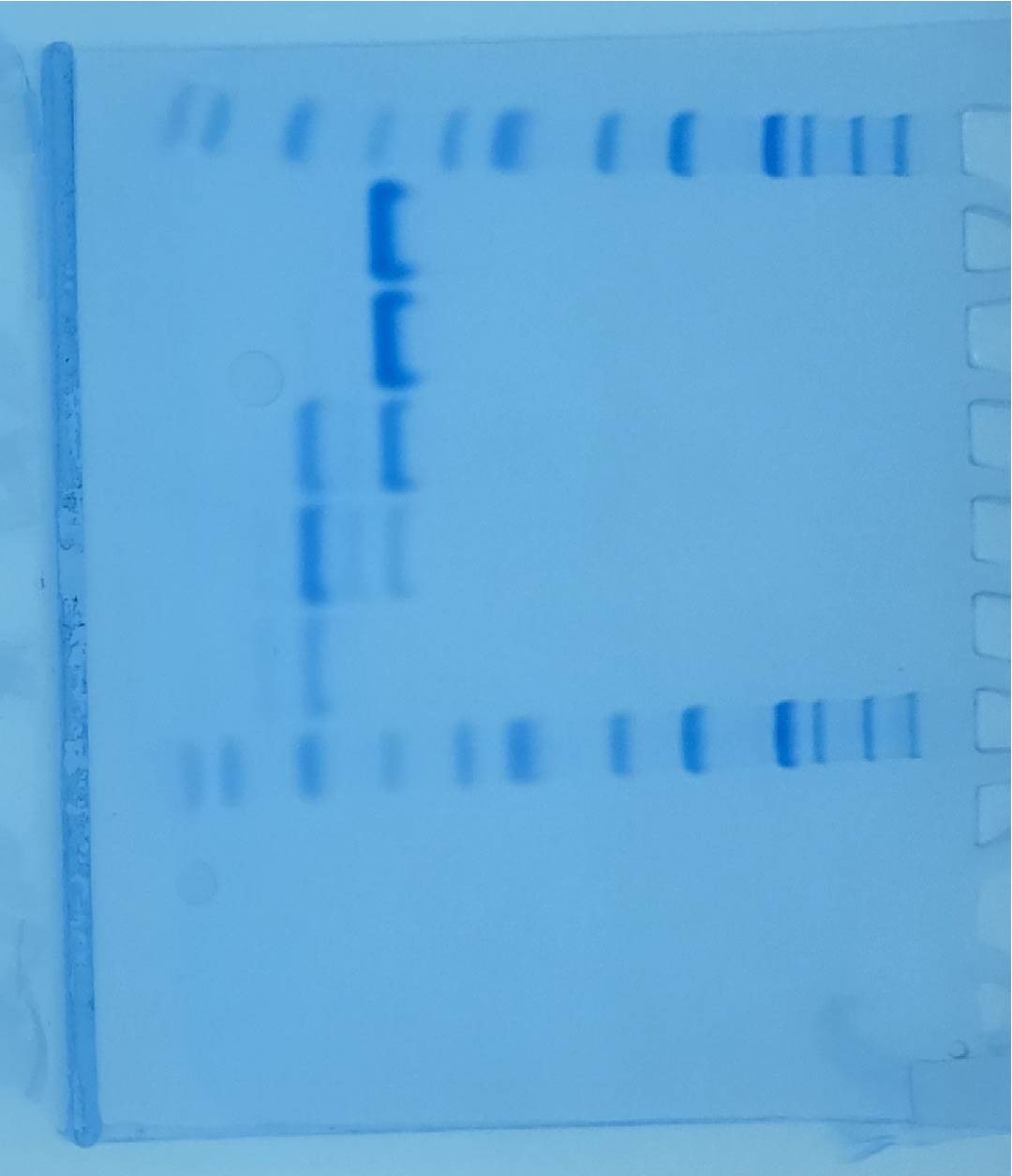

Supplement: Supplementary file 4 — Source Data [file 41467_2025_58899_MOESM4_ESM.zip › Full gel of Supplementary Figure 1c.jpg]

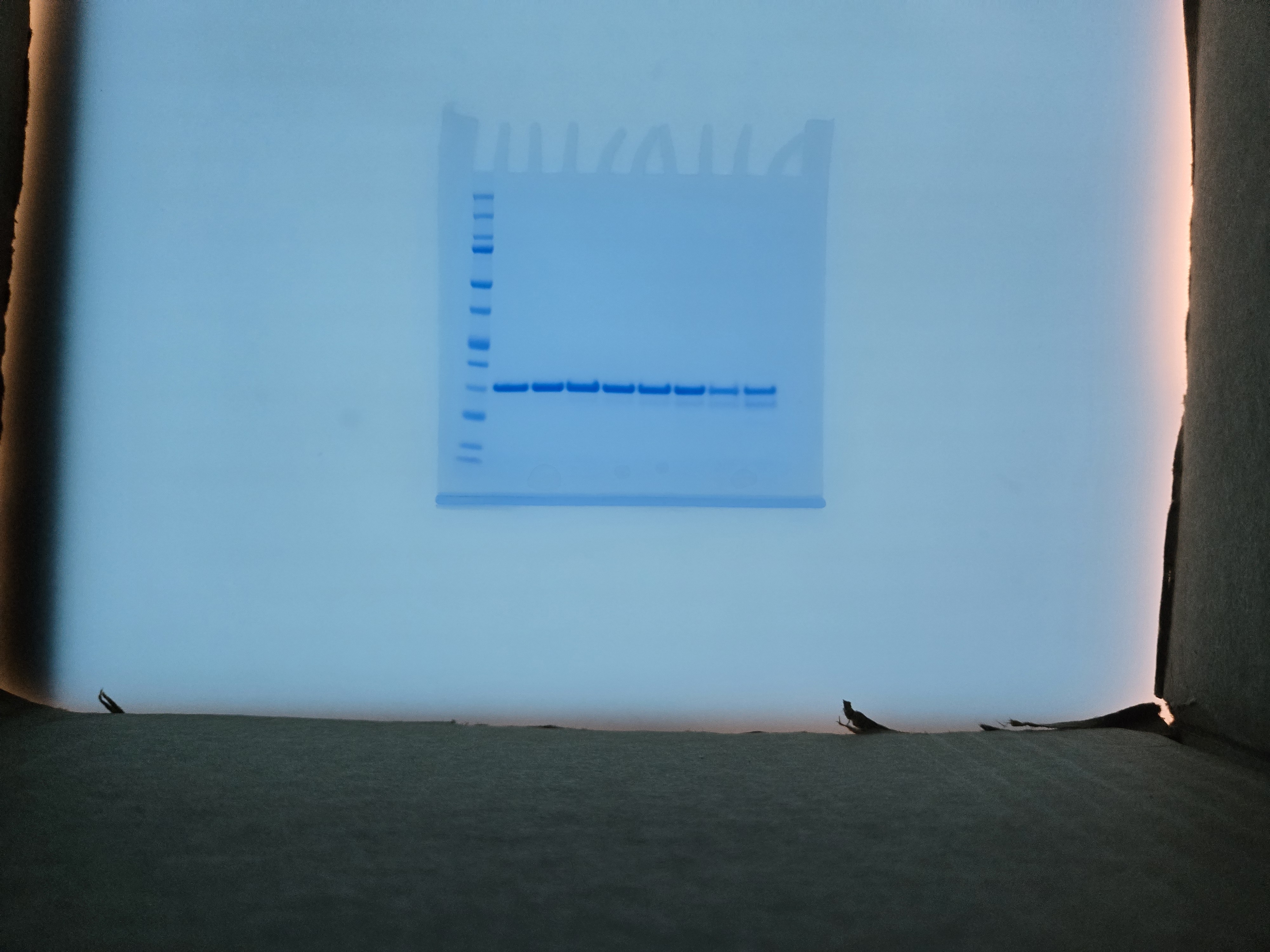

Supplement: Supplementary file 4 — Source Data [file 41467_2025_58899_MOESM4_ESM.zip › Full gel of Supplementary Figure 1d.jpg]
